# Amino acid- and lipid-related metabolic remodeling in PTZ-kindled mice reveals candidate plasma signatures of chronic epilepsy

**DOI:** 10.3389/fnins.2026.1889410

**Published:** 2026-08-20

**Authors:** Juan Ren, Fuli Wang, Zijian Li, Zhen Liang, Songyan Liu

**Affiliations:** Department of Neurology, China-Japan Union Hospital of Jilin University, Changchun, Jilin, China

**Keywords:** epilepsy, indolelactic acid, lipid metabolism, metabolomics, PTZ-kindled mouse model, translational neuroscience, tryptophan metabolism

## Abstract

**Background:**

Metabolic dysfunction is increasingly implicated in epilepsy, but the systemic metabolic alterations associated with chronic seizures remain incompletely characterized. This study aimed to define peripheral metabolic signatures of chronic epilepsy in a pentylenetetrazol (PTZ)-kindled mouse model and to examine whether the altered pathways were supported by human epilepsy transcriptomic data.

**Methods:**

Male C57BL/6 mice were subjected to repeated PTZ injections to establish chronic epilepsy, while control mice received saline. Plasma samples were analyzed using ultra-high-performance liquid chromatography-mass spectrometry-based untargeted metabolomics. Differential metabolites were identified through multivariate and univariate analyses, followed by KEGG pathway enrichment. Least absolute shrinkage and selection operator (LASSO) regression was used as an exploratory feature-selection method. To provide complementary human hippocampal transcriptomic context, the hippocampal subset of GSE256068 was reanalyzed using differential expression analysis, and KEGG pathway enrichment analysis.

**Results:**

PTZ-kindled mice displayed a distinct plasma metabolic profile compared with controls. A total of 349 differential metabolites were identified, mainly enriched in amino acid metabolism, tryptophan metabolism, and central carbon metabolism. LASSO regression identified six candidate metabolic features, all of which are reported in the supplementary material. Among them, indolelactic acid (ILA) and DG (20:3n6/0:0/20:3n6) were prioritized for biological interpretation because of their relevance to tryptophan-related and lipid-related metabolism. Reanalysis of the human hippocampal transcriptomic dataset revealed pathway-level alterations related to GABAergic synaptic signaling, glycosphingolipid biosynthesis, inflammatory regulation of TRP channels, and inflammatory response, providing complementary human hippocampal context for the neurotransmitter-, lipid-, and inflammatory--related metabolic alterations observed in PTZ-kindled mice.

**Conclusion:**

PTZ-induced kindling was associated with marked plasma metabolic alterations involving amino acid-, tryptophan-, and lipid-related pathways. ILA and DG (20:3n6/0:0/20:3n6) emerged as candidate plasma metabolic features associated with the PTZ-kindled seizure phenotype. Complementary analysis of human temporal lobe epilepsy with hippocampal sclerosis (TLE-HS) hippocampal transcriptomic data identified related alterations in neurotransmission-, lipid-, and inflammatory-associated pathways. This pathway-level convergence supports the broader relevance of metabolic dysregulation to epilepsy, while the mechanistic roles and biomarker potential of individual metabolites require independent validation.

## 1 Introduction

Epilepsy is a common neurological disorder characterized clinically by recurrent spontaneous seizures caused by abnormal neuronal activity in the brain (Krishnamurthy, 2025). Although numerous antiseizure medications are currently available, nearly one-third of patients continue to suffer from drug-resistant seizures, indicating that the biological mechanisms underlying epileptogenesis remain incompletely understood (Löscher and Klein, 2021). In recent years, metabolic disturbances have been increasingly recognized as important contributors to epilepsy. Because neuronal activity depends on tightly regulated metabolic processes and a continuous energy supply, alterations in metabolic homeostasis may influence neuronal excitability, synaptic transmission, and neuroinflammatory responses that participate in seizure generation (Krishnamurthy, 2025; Löscher and Klein, 2021).

Metabolomics provides a powerful analytical approach for systematically characterizing small-molecule metabolites in biological systems. By capturing global metabolic changes associated with disease states, metabolomic profiling can reveal altered metabolic pathways and facilitate the discovery of candidate biomarkers (Zhou et al., 2023). Previous metabolomic investigations have reported widespread metabolic alterations in epilepsy, particularly in pathways related to amino acid metabolism, lipid metabolism, and energy metabolism (Rho and Boison, 2022; Zhou et al., 2023; Lian et al., 2024).

Among these pathways, amino acid metabolism is closely linked to neuronal signaling because several amino acids serve as precursors or regulators of neurotransmitters. In the central nervous system, glutamate and *γ*-aminobutyric acid (GABA) function as the primary excitatory and inhibitory neurotransmitters, respectively. Maintaining the balance between these two systems is essential for normal neuronal activity, and disruption of this equilibrium has long been associated with seizure initiation and propagation (Perucca et al., 2023a). Notably, tryptophan metabolism has also attracted considerable attention in studies of neurological disorders. As an essential amino acid, tryptophan can be processed through several metabolic routes, including the kynurenine pathway, the serotonin pathway, and microbial indole pathways. Metabolites generated through these pathways participate in multiple physiological processes, such as neurotransmitter regulation, immune responses, oxidative stress, and neuroinflammation (Agus et al., 2018; Xia and Huang, 2026; Cerdán-Centeno et al., 2026; Shaw et al., 2023).

In addition to host metabolic pathways, the gut microbiota can influence systemic metabolism by transforming dietary and endogenous substrates into bioactive compounds. Some indole derivatives generated from tryptophan metabolism have been reported to be associated with microbial metabolism and may enter the circulation. Previous studies have suggested that microbiota-associated metabolic alterations may be relevant to epilepsy (Agus et al., 2018; Olasunkanmi et al., 2026; Ding et al., 2021; Li et al., 2025; Bian and Shao, 2024; Yan et al., 2025). However, because microbiota composition and intestinal function were not directly assessed in the present study, microbiota-related interpretations should be considered hypothesis-generating rather than mechanistically demonstrated.

In the present study, we used an untargeted ultra-high-performance liquid chromatography-mass spectrometry (UHPLC–MS)-based metabolomics approach to characterize plasma metabolic alterations in a PTZ-kindled mouse model of chronic epilepsy. We further applied exploratory feature-selection analysis to prioritize candidate metabolites associated with the PTZ-kindled seizure phenotype. To provide complementary human transcriptomic context, we reanalyzed the hippocampal subset of a public temporal lobe epilepsy transcriptomic dataset at the pathway level. We hypothesized that PTZ-induced kindling is associated with coordinated alterations in amino acid-, tryptophan-, and lipid-related metabolic pathways, some of which may also be implicated in human epilepsy.

## 2 Materials and methods

### 2.1 Animals and experimental design

Male C57BL/6 mice were used in this study and randomly divided into two groups: the epilepsy group (PTZ, *n* = 6) and the control group (CON, *n* = 6). All animals were housed under standard laboratory conditions with a controlled temperature, humidity, and a 12 h light/dark cycle, with free access to food and water. All experimental procedures were performed in accordance with institutional guidelines for the care and use of laboratory animals. All animal experiments were conducted in accordance with institutional guidelines and were approved by the Animal Care Welfare Committee of Jilin University (approval no. KT202603258).

To establish a PTZ-induced kindling model, mice in the epilepsy group were intraperitoneally injected with PTZ at a dose of 35 mg/kg every other day for a total of 11 injections over 21 days. Mice in the control group received equal volumes of normal saline following the same schedule (El-Sayed et al., 2023).

### 2.2 Behavioral assessment of seizures

After each PTZ injection, mice were individually observed for seizure behaviors for half an hour and scored according to the Racine scale. The seizure severity was classified into six stages: (I) facial automatisms and rhythmic head nodding; (II) head clonus with neck muscle involvement; (III) unilateral forelimb clonus; (IV) bilateral forelimb clonus with rearing; (V) rearing and falling accompanied by generalized tonic–clonic convulsions; and (VI) loss of posture with continuous jumping, wild running, or death.

The maximum Racine score during the observation period was recorded after each PTZ injection. Latency to generalized seizure was defined as the time from completion of PTZ injection to the onset of the first Racine stage IV or higher seizure. Seizure duration was defined as the time from onset of Racine stage IV or higher seizure behavior to recovery of normal posture and spontaneous locomotion. Successful PTZ kindling was defined as the occurrence of Racine stage IV or higher seizures for at least three consecutive stimulations, consistent with the predefined criterion (Yang J. et al., 2024). Individual seizure phenotype data for the six PTZ-treated mice included in metabolomics analysis are provided in Supplementary Table S1.

### 2.3 Peripheral blood sample collection

Peripheral blood samples were collected approximately 24 h after the final PTZ injection. Before sample collection, mice were fasted for 6–8 h. Following euthanasia, peripheral blood was collected by orbital enucleation and immediately transferred into EDTA-containing tubes. After collection, the tubes were gently inverted 8–10 times to ensure adequate mixing of blood with the anticoagulant.

The collected whole blood samples were centrifuged at 3,000 rpm for 10 min at 4 °C, resulting in separation into three layers: plasma (upper layer), buffy coat (middle layer), and erythrocytes (lower layer). The plasma layer was carefully transferred to a new centrifuge tube and further centrifuged at 3,000 rpm for 10 min at 4 °C to remove residual cellular debris. The resulting supernatant was then carefully transferred to clean EP tubes and stored at −80 °C until subsequent metabolomic analysis.

### 2.4 Untargeted metabolomics analysis

#### 2.4.1 Sample preparation

Plasma samples were thawed at 4 °C prior to metabolite extraction. For each sample, 100 μL of plasma was transferred into an EP tube, and 700 μL of extraction solvent containing internal standards (methanol: acetonitrile: water = 4:2:1, v/v/v) was added. The internal standards included d3-leucine, 13C9-phenylalanine, d5-tryptophan, and 13C3-progesterone. Samples were vortexed for 1 min and incubated at −20 °C for 2 h to precipitate proteins. The mixture was centrifuged at 25,000 g at 4 °C for 15 min, and 600 μL of the supernatant was transferred to a new tube and dried using a vacuum concentrator. The dried extracts were reconstituted in 180 μL methanol: water (1:1, v/v), vortexed for 10 min, and centrifuged again at 25,000 g for 15 min at 4 °C. The supernatant was collected for LC–MS analysis. Quality control (QC) samples were prepared by pooling equal volumes from each sample.

#### 2.4.2 UHPLC–MS analysis

Metabolomic profiling was performed using an ultra-high-performance liquid chromatography system coupled with a Q Exactive mass spectrometer (Thermo Fisher Scientific, USA). Data acquisition was conducted in both positive and negative electrospray ionization modes. The full scan range was set from m/z 70 to 1,050 with a resolution of 70,000. The automatic gain control (AGC) target for MS acquisition was set to 3 × 10^6^ with a maximum ion injection time of 100 ms. The top three precursor ions were selected for MS/MS fragmentation with a resolution of 17,500, AGC target of 1 × 10^5^, and maximum ion injection time of 50 ms. Stepped normalized collision energies were set to 20, 40, and 60 eV.

The electrospray ionization parameters were as follows: sheath gas flow rate of 40, auxiliary gas flow rate of 10, spray voltage of 3.80 kV in positive mode and 3.20 kV in negative mode, capillary temperature of 320 °C, and auxiliary gas heater temperature of 350 °C.

#### 2.4.3 Metabolite identification and data preprocessing

Raw mass spectrometry data were processed using Compound Discoverer software (version 3.3, Thermo Fisher Scientific, USA) for peak extraction, alignment, and metabolite identification. Metabolite annotation was performed by comparing spectra with the BMDB (BGI Metabolome Database), mzCloud, and ChemSpider databases. Identification parameters included a parent ion mass deviation of less than 5 ppm, fragment ion mass deviation of less than 10 ppm, and retention time deviation within 0.2 min.

The resulting metabolite data matrix was further processed using MetaX software for normalization and batch correction. Probabilistic quotient normalization (PQN) was applied to normalize the data, and quality control-based robust LOESS signal correction (QC-RLSC) was used to correct batch effects. Metabolites with a coefficient of variation greater than 30% in QC samples were removed (Tseng et al., 2025; Hughes et al., 2025; Leach et al., 2023).

#### 2.4.4 Multivariate statistical analysis

Principal component analysis (PCA) was used as an unsupervised method to evaluate sample clustering and data quality. Log transformation and Pareto scaling were applied before analysis. Partial least squares discriminant analysis (PLS-DA) and orthogonal partial least squares discriminant analysis (OPLS-DA) were performed to explore metabolic differences between groups. The variance explained by each component for PCA, PLS-DA, and OPLS-DA models is provided in Supplementary Table S2. Variable importance in projection (VIP) values were calculated to estimate the contribution of each metabolite to group discrimination. Metabolites with VIP ≥ 1, *p* < 0.05 (two-tailed Student’s *t*-test), and fold change (FC) ≥ 1.2 or ≤0.83 were considered significantly different between groups (Wang et al., 2022).

#### 2.4.5 Pathway enrichment analysis

Functional annotation of identified metabolites was performed using the Human Metabolome Database (HMDB) and the Kyoto Encyclopedia of Genes and Genomes (KEGG) database. KEGG pathway enrichment analysis was conducted to identify significantly altered metabolic pathways associated with epilepsy.

### 2.5 Exploratory feature-selection analysis

Least absolute shrinkage and selection operator (LASSO) logistic regression was applied to the differentially abundant metabolites as an exploratory feature-selection analysis. Group membership was coded as PTZ = 0 and CON = 1. The optimal penalty parameter was selected by 10-fold cross-validation using the value that minimized the mean cross-validated binomial deviance (λmin). Metabolites with nonzero coefficients at λmin were retained as candidate metabolic features. Under this coding scheme, a negative coefficient indicated that a higher relative abundance was associated with the PTZ group. Given the limited sample size, the LASSO analysis was considered exploratory (Chen et al., 2024).

Possible origins or previously reported associations of the LASSO-selected features were evaluated using metabolite databases and published literature. These annotations were used solely to facilitate biological interpretation and were not applied as an additional statistical exclusion criterion or used to refit the LASSO model. The relative abundances of features prioritized for biological interpretation were visualized using boxplots and compared between the PTZ and CON groups using the Mann–Whitney U test. All tests were two-sided, and *p* < 0.05 was considered statistically significant.

### 2.6 Complementary human hippocampal transcriptomic analysis

To provide complementary human hippocampal transcriptomic context for the metabolomic findings, the publicly available transcriptomic dataset GSE256068 was obtained from the Gene Expression Omnibus (GEO) database (François et al., 2024). To reduce heterogeneity associated with brain region and disease subtype, the analysis was restricted to 59 hippocampal samples from patients with drug-resistant temporal lobe epilepsy with hippocampal sclerosis (TLE-HS) and 11 control hippocampal samples. The TLE-HS samples were surgically resected tissues, whereas the control samples were postmortem tissues from individuals without a history of seizures or other neurological diseases. Available sample-level information, including age, sex, brain region, disease subtype, group assignment, and seizure frequency, is summarized in Supplementary Table S5.

Raw gene-count data and sample metadata for GSE256068 were obtained from the GEO database. The analysis was restricted to 59 TLE-HS samples and 11 control hippocampal samples. Genes with counts of at least 10 in three or more samples were retained. Differential-expression analysis between the TLE-HS and control hippocampal groups was performed in R version 4.5.1 using DESeq2, with group included as the explanatory variable and the control hippocampal group set as the reference. *p* values were adjusted for multiple testing using the Benjamini–Hochberg method. Positive log_2_FC values indicated higher expression in the TLE-HS group, whereas negative values indicated lower expression. Genes with an adjusted *p* value < 0.05 and an absolute log_2_FC > 0.585 were classified as differentially expressed.

Downstream analyses were performed in R version 4.5.1 using the tidyverse, ggrepel, clusterProfiler, org.Hs.eg.db, and enrichplot packages. For Kyoto Encyclopedia of Genes and Genomes (KEGG) over-representation analysis, the gene symbols of significant differentially expressed genes were converted to Entrez gene identifiers using org. Hs.eg.db. KEGG pathway enrichment analysis was then performed using the enrichKEGG function in clusterProfiler, with *Homo sapiens* specified as the organism.

As a descriptive gene-level analysis, representative genes were selected from the key metabolic pathways identified in the mouse plasma metabolomic analysis: PHGDH and PSAT1 (serine biosynthesis, mapping to the enriched amino acid metabolism pathways), GAD1 (glutamate–GABA conversion, mapping to neurotransmitter-related amino acid metabolism), FASN (fatty acid synthesis, mapping to the lipid-related metabolic alterations), and TDO2 (tryptophan 2,3-dioxygenase, mapping to the enriched tryptophan metabolism pathway). Variance-stabilized expression values for these genes were extracted from the DESeq2-processed GSE256068 expression matrix. Entrez gene identifiers were mapped to gene symbols using org. Hs.eg.db. The expression values were visualized using boxplots in GraphPad Prism (version 10.1.2). The log_2_FC and adjusted *p* values for these genes were obtained from the formal differential-expression analysis of the same TLE-HS and control hippocampal samples; no additional statistical tests were applied to the plotted expression values. These gene-level findings were considered descriptive and are presented in Supplementary Figure S1 and Supplementary Table S6.

## 3 Results

### 3.1 Establishment of the PTZ-induced epilepsy mouse model

**Figure 1:**
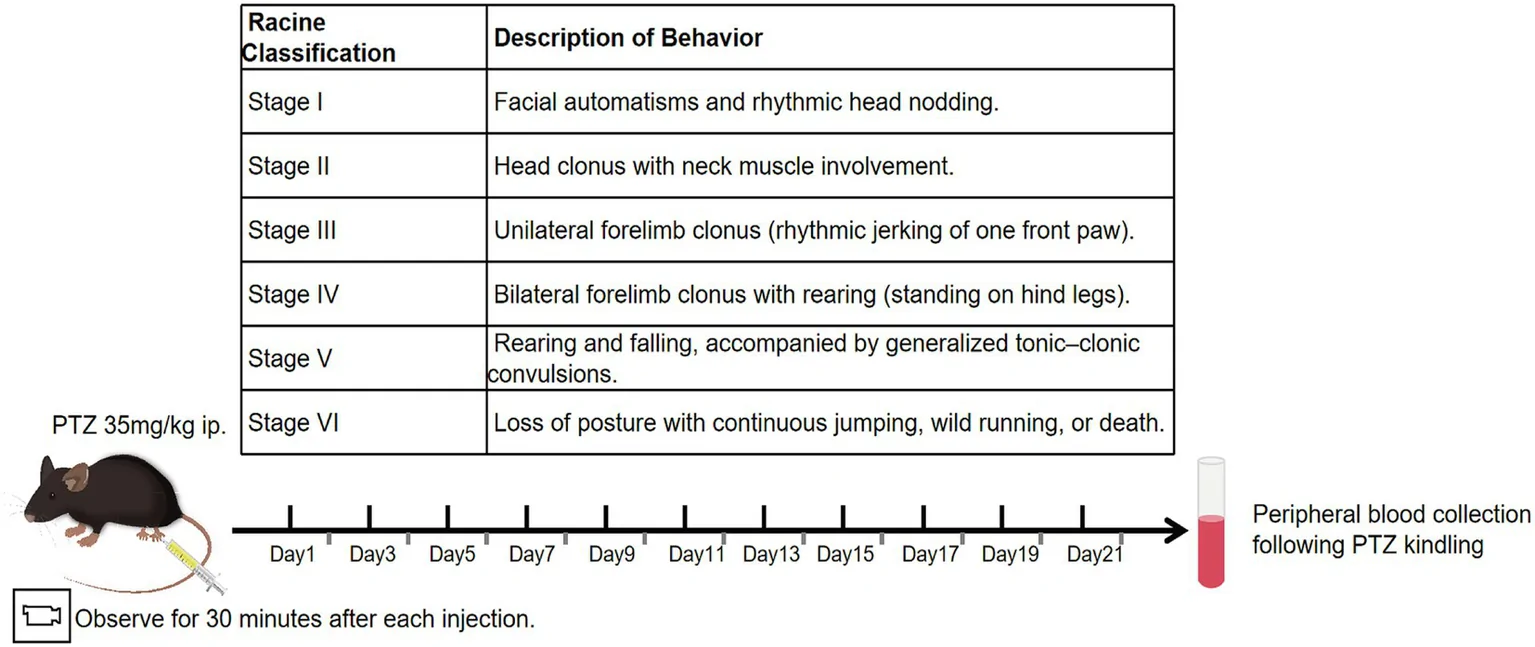
Experimental design for establishing chronic PTZ-kindling mouse model of epilepsy.

Figure 1 illustrates the experimental design of the PTZ kindling model, including the intraperitoneal injection schedule, sampling timeline, and Racine scale used for behavioral seizure scoring. Mice in the PTZ group received repeated PTZ administration, while control mice received saline according to the same timeline. Seizure behaviors were evaluated using the Racine classification criteria shown in Figure 1. Of the eight PTZ-treated mice, six met the predefined kindling criterion (≥3 consecutive stimulations with Racine stage IV or higher) and were included in the metabolomic analysis. One mouse died during the kindling procedure, and one was excluded due to failure to meet the kindling criterion. Individual seizure phenotype data for the six included mice are provided in Supplementary Table S1.

### 3.2 Overview of metabolomic profiling and data quality assessment

Untargeted metabolomic profiling of plasma samples was performed using UPLC–MS. A total of 13,608 metabolite features were detected across all samples after data preprocessing and quality filtering.

**Figure 2:**
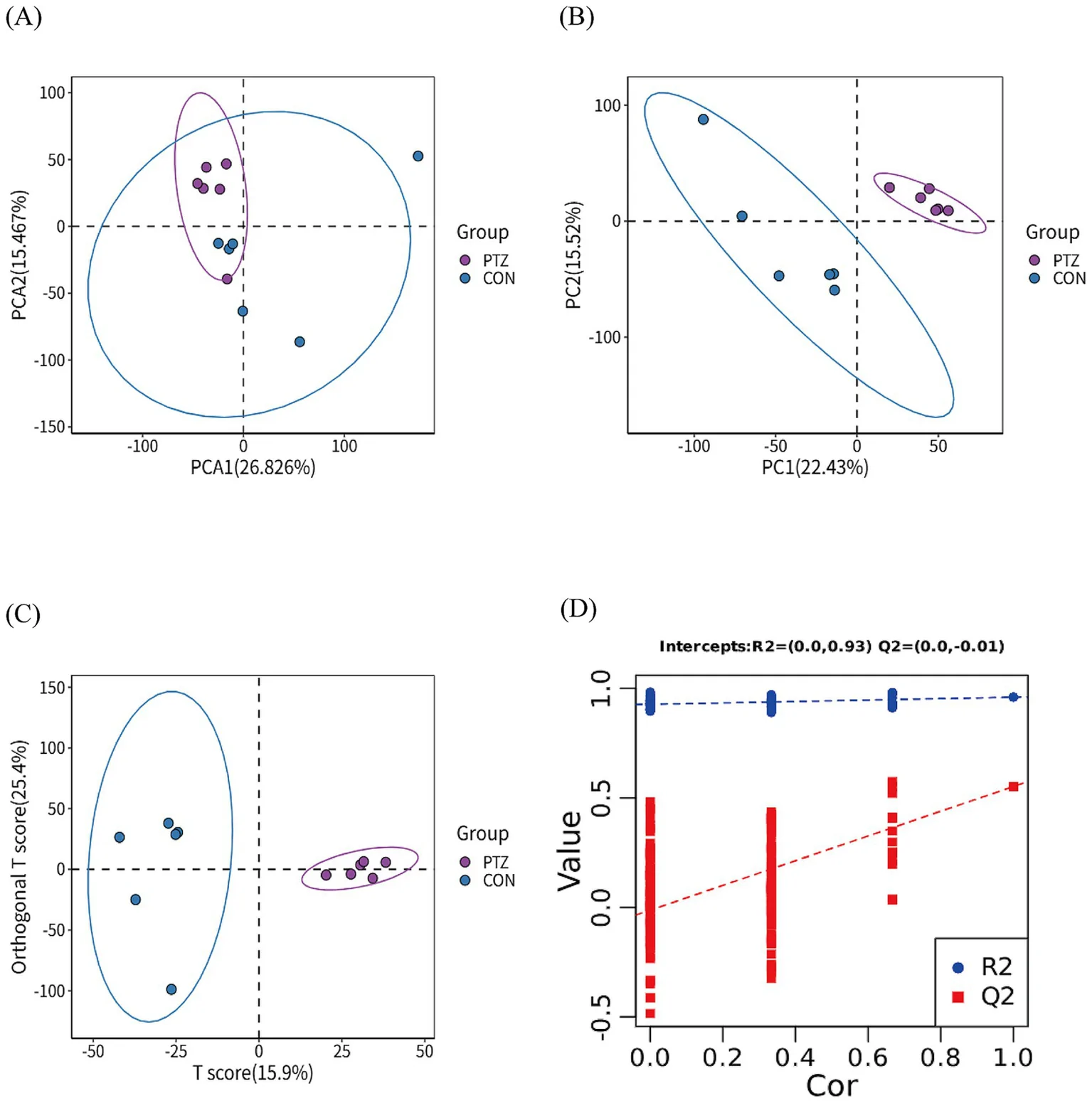
Multivariate statistical analysis of metabolomics data between PTZ and CON groups. **(A)** PCA score plot. Unsupervised analysis showing sample distribution between PTZ and CON groups, ellipses represent 95% confidence intervals; **(B)** PLS-DA score plot showing the separation trend between the two groups; **(C)** OPLS-DA score plot further confirming the metabolic differences between groups; **(D)** Permutation test of the PLS-DA model (200 permutations), with an R^2^ intercept of 0.93 and a Q^2^ intercept of −0.01, indicating no overfitting.

Principal component analysis (PCA) was conducted to evaluate the overall distribution of the samples and the stability of the analytical platform. Moreover, a separation trend between the epilepsy group and the control group was observed in the PCA score plot (Figure 2A), suggesting the presence of metabolic differences between the two groups.

### 3.3 Multivariate analysis reveals metabolic differences between groups

To further investigate metabolic differences between the epilepsy and control groups, supervised multivariate analyses were performed. Partial least squares discriminant analysis (PLS-DA) showed a clear separation between the two groups (Figure 2B), indicating distinct metabolic profiles.

Orthogonal partial least squares discriminant analysis (OPLS-DA) further confirmed the metabolic differences between groups (Figure 2C). The model parameters demonstrated good explanatory and predictive ability (R2Y = 0.96, Q2 = 0.552). Permutation testing yielded an R2 intercept of 0.93 and a Q2 intercept of −0.01 (Figure 2D).

### 3.4 Identification of differential metabolites

Differential metabolites between the epilepsy and control groups were screened based on the criteria of VIP ≥ 1, *p* < 0.05, and absolute FC ≥ 1.2 (i.e., FC ≥ 1.2 or FC ≤ 0.83). A total of 349 metabolites were identified as significantly altered, including 183 upregulated metabolites and 166 downregulated metabolites. The complete list of the 349 significantly altered metabolites is provided in Supplementary Table S3.

**Figure 3:**
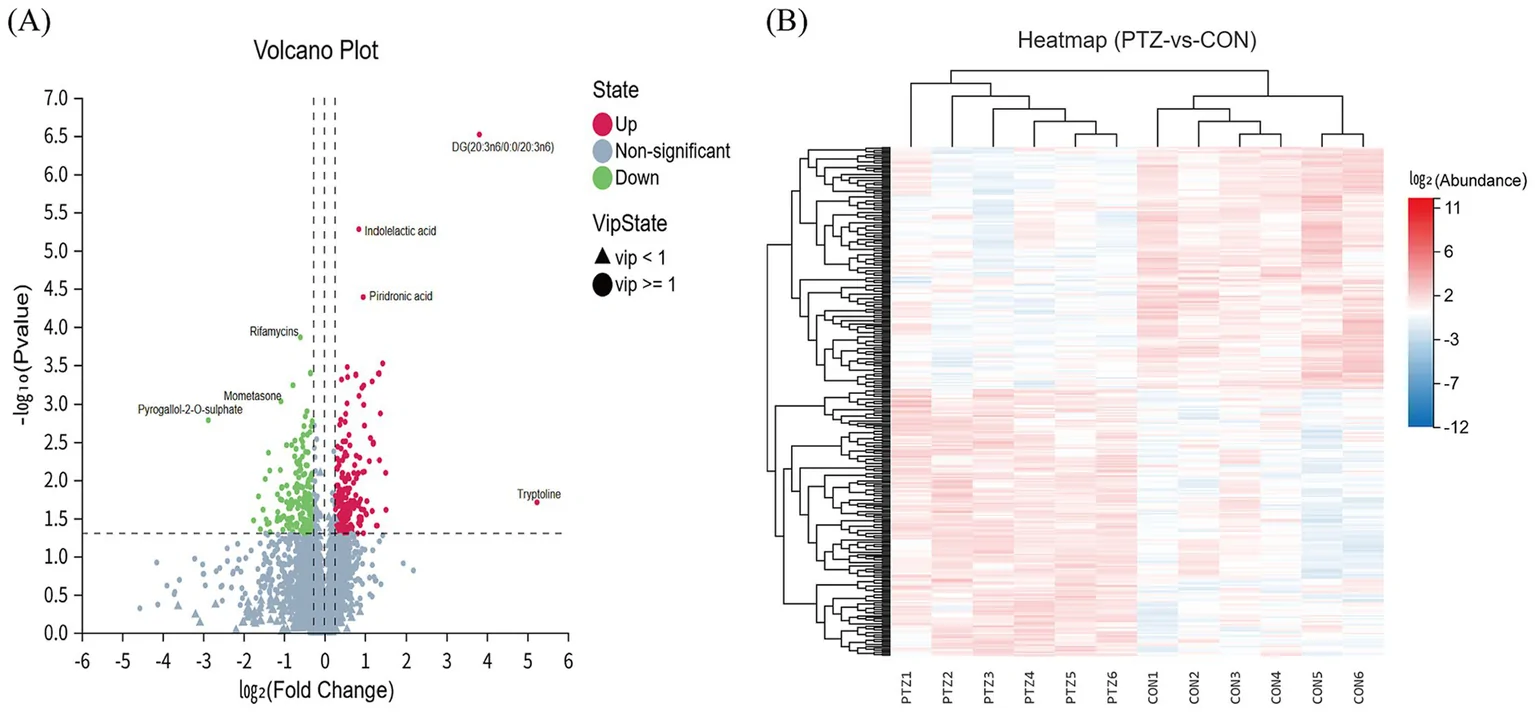
Identification of differential metabolites between PTZ and CON groups. **(A)** Volcano plot. Red dots indicate significantly upregulated metabolites (VIP ≥ 1, *p* < 0.05), green dots indicate significantly downregulated metabolites, and blue dots indicate non-significant metabolites. Metabolites with VIP ≥ 1 are marked with black circles, while those with VIP < 1 are marked with black triangles. **(B)** Heatmap. Hierarchical clustering heatmap showing the relative abundance of differential metabolites. Each column represents an individual sample (PTZ1-6, CON1-6), and each row represents a metabolite. The color scale represents log_2_ abundance, with red (+11) indicating high abundance and blue (−12) indicating low abundance.

A volcano plot was constructed to visualize the distribution of differential metabolites, with key metabolites-including the top upregulated and downregulated features and LASSO-selected candidates (ILA and DG)-explicitly labeled (Figure 3A). In addition, hierarchical clustering analysis showed distinct metabolic patterns between the two groups (Figure 3B).

**Figure 4:**
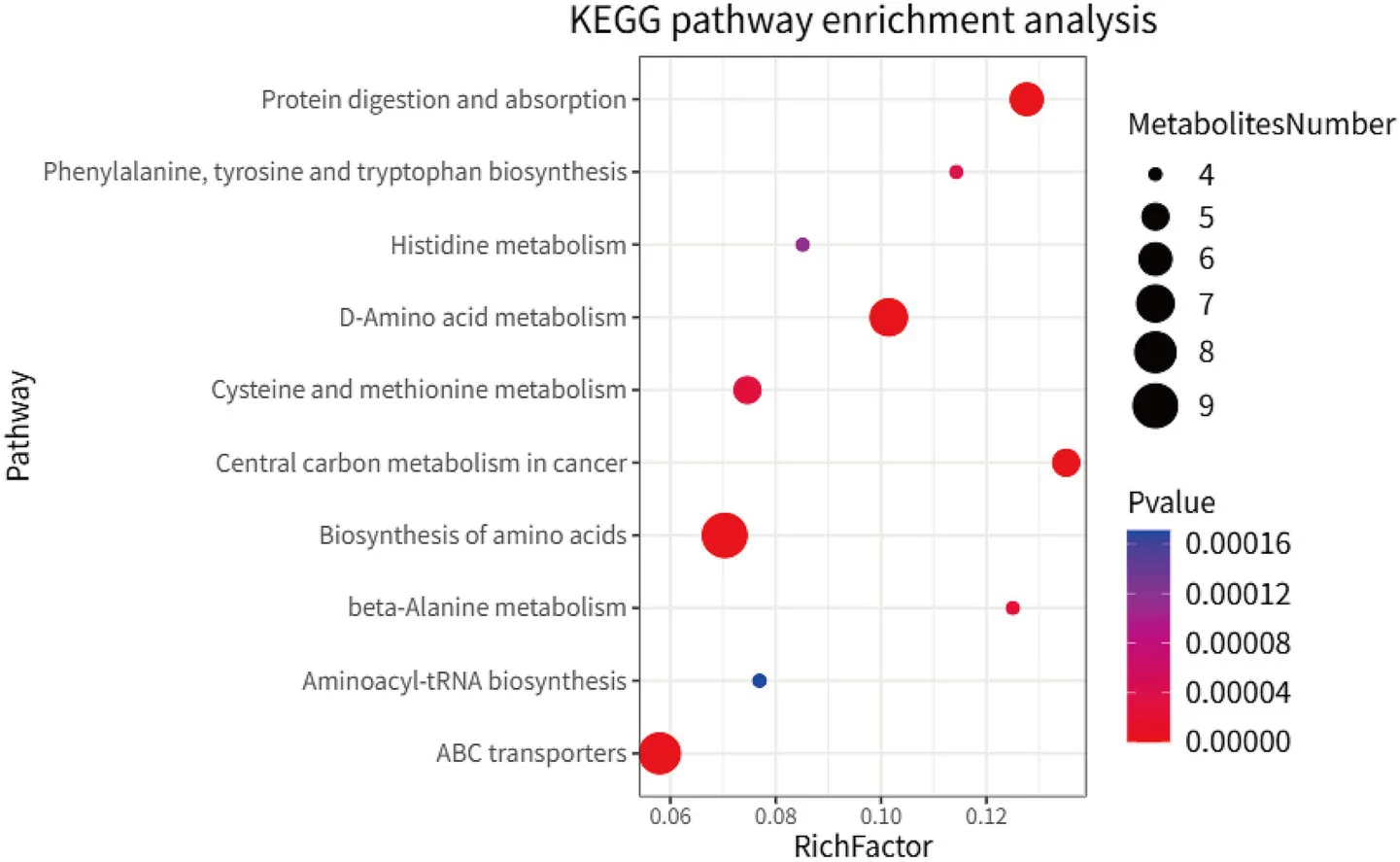
KEGG pathway enrichment analysis of differential metabolites. The x-axis shows the rich factor. Bubble size indicates the number of enriched metabolites, and bubble color represents the *p* value.

To explore the biological significance of the differential metabolites, KEGG pathway enrichment analysis was performed. As shown in Figure 4, the differential metabolites were mainly enriched in pathways related to amino acid metabolism, tryptophan metabolism, and central carbon metabolism.

These pathways are closely associated with neurotransmitter synthesis, energy metabolism, and neuroinflammatory responses, suggesting that these pathways are associated with the PTZ-kindled metabolic phenotype.

### 3.5 Exploratory LASSO analysis identifies candidate metabolic features

**Figure 5:**
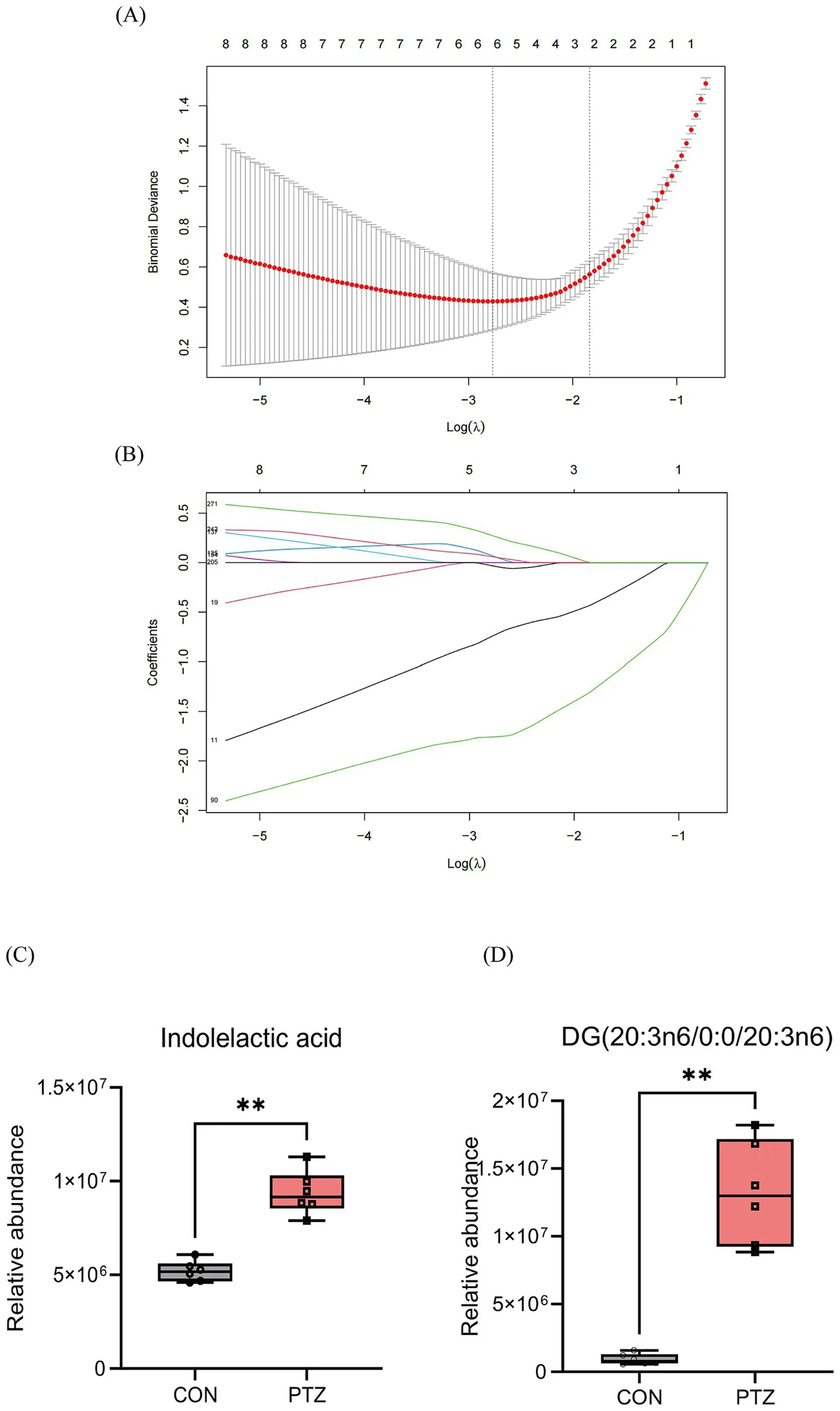
LASSO regression-based feature selection and validation of candidate biomarkers. **(A)** Cross-validation deviance plot. Binomial deviance as a function of log(*λ*). The vertical dashed line indicates *λ*.min = 0.0628, where the deviance reaches its minimum (0.43). **(B)** LASSO coefficient paths. As log(*λ*) increases, most coefficients are compressed toward zero. At *λ*.min, six variables retained non-zero coefficients. **(C)** Boxplot of indolelactic acid (ILA). Comparison of ILA levels between PTZ and CON groups (Mann–Whitney U test). **(D)** Boxplot of DG (20:3n6/0:0/20:3n6). Comparison of DG (20:3n6/0:0/20:3n6) levels between PTZ and CON groups (Mann–Whitney U test). *p < 0.05; **p < 0.01; ***p < 0.001; ****p < 0.0001.

LASSO logistic regression was performed to identify metabolic features associated with the PTZ-kindled state. The optimal penalty parameter was determined using 10-fold cross-validation, and the minimum binomial deviance was observed at *λ* = 0.0628 (Figure 5A). Figure 5B shows the corresponding coefficient paths as a function of log(λ), where most coefficients were gradually shrunk to zero as λ increased. At this value, six features had nonzero coefficients: indolelactic acid (ILA), DG (20:3n6/0:0/20:3n6), Rifamycins, 3-oxobrimonidine, Pyrogallol-2-O-sulfate, and Mometasone. All six LASSO-selected features are reported in Supplementary Table S4.

Among these variables, ILA (coefficient = −0.7360) and DG (20:3n6/0:0/20:3n6) (coefficient = −1.7579) were prioritized for biological interpretation because of their relevance to tryptophan-related and lipid-related metabolism, respectively. The other LASSO-selected features were retained in the Supplementary Table S4, and their possible origins or reported associations were annotated based on available databases and published literature. This annotation was used to aid biological interpretation and was not treated as an additional statistical exclusion step.

Boxplots further showed that both metabolites were significantly increased in the PTZ-kindled epilepsy model group compared with the control group (Figures 5C,D). The negative coefficients reflect the coding scheme of the logistic regression model (PTZ = 0, CON = 1) rather than a decrease in metabolite abundance. These findings suggest that ILA and a DG species may represent potential metabolic signatures associated with the PTZ-kindled state.

### 3.6 Human hippocampal transcriptomic analysis provides complementary pathway-level context

To provide human transcriptomic context for the metabolic alterations identified in this study, the publicly available GSE256068 dataset from patients with TLE-HS was analyzed. Differential-expression and KEGG pathway enrichment analyses identified alterations in multiple epilepsy- and metabolism-related biological processes, including GABAergic synapse signaling, glycosphingolipid biosynthesis, inflammatory mediator regulation of TRP channels, TNF signaling, and NF-kappa B signaling (Supplementary Figure S2; Supplementary Table S7). In particular, the enrichment of GABAergic synapse-related genes provided complementary context for the alterations in amino acid and neurotransmitter-related metabolism observed in the mouse plasma metabolomic analysis.

Several genes representing the key metabolic pathways identified in the mouse metabolomic analysis—including PHGDH and PSAT1 (serine biosynthesis), GAD1 (glutamate–GABA metabolism), FASN (fatty acid synthesis), and TDO2 (tryptophan–kynurenine metabolism)—showed differential expression between TLE-HS patients and controls (Supplementary Figure S1; Supplementary Table S6). All five genes were significantly upregulated in TLE-HS hippocampus (*p* < 0.05, DESeq2). Together, these findings suggest that epilepsy may involve alterations in multiple metabolic pathways, including amino acid metabolism, tryptophan metabolism, and lipid metabolism.

At a descriptive level, the human transcriptomic results showed partial pathway-level parallels with the metabolomic findings obtained from the PTZ-kindled mouse model. The parallel observations in the experimental model and human TLE-HS samples suggest the potential relevance of metabolic dysregulation to epilepsy; however, they should not be interpreted as direct cross-species validation.

### 3.7 Summary framework of metabolomic and transcriptomic pathway alterations

**Figure 6:**
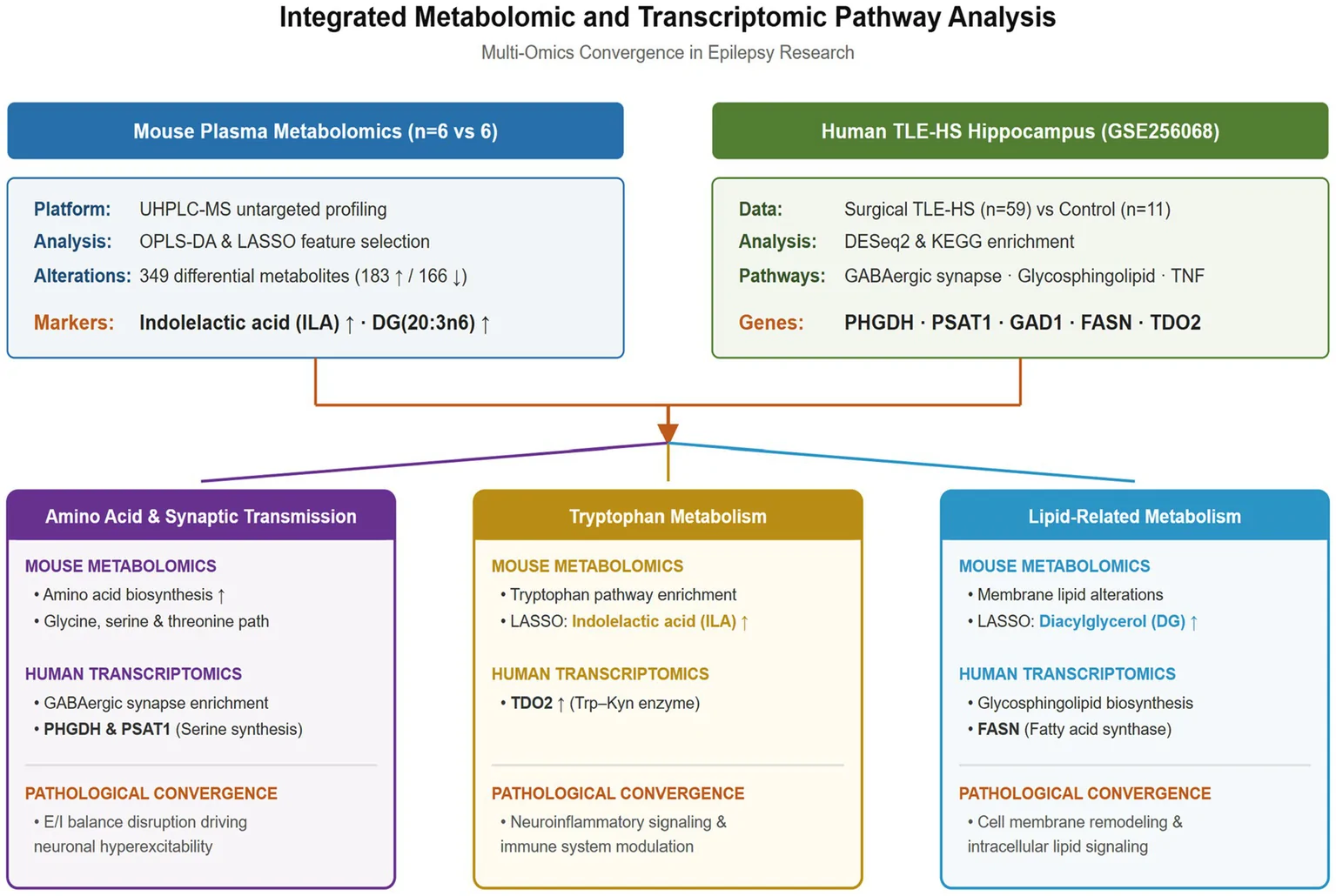
Integrated metabolomic and transcriptomic network of epilepsy-associated metabolic pathways.

To summarize the metabolomic findings together with complementary external transcriptomic evidence, we constructed a pathway-level summary framework integrating LC–MS metabolomics data from the PTZ-kindled mouse model and hippocampal transcriptomic pathway results from GSE256068 (Figure 6). This framework highlighted three major axes associated with the PTZ-kindled metabolic phenotype: amino acid/neurotransmitter-related metabolism, tryptophan-related metabolism, and lipid-related metabolism.

In the amino acid /neurotransmitter-related branch, altered plasma metabolites and hippocampal transcriptomic enrichment of GABAergic synapse suggested involvement of neurotransmission-related pathways. In the lipid-related branch, altered DG abundance and enrichment of glycosphingolipid biosynthesis suggested involvement of lipid-associated membrane metabolism. In the tryptophan-related branch, increased ILA suggested altered tryptophan-related metabolism. Because gut microbiota composition was not measured, the microbiota-related interpretation of ILA remains hypothesis-generating.

Overall, this framework provides a descriptive pathway-level summary of seizure-associated metabolic and transcriptomic alterations. It should not be interpreted as direct metabolite-gene correlation or direct cross-species validation.

## 4 Discussion

In this study, untargeted LC–MS metabolomics revealed broad alterations in the plasma metabolic profile of PTZ-kindled mice. Multivariate analyses indicated group-level separation between PTZ-kindled and control mice, suggesting that repeated PTZ exposure and the resulting kindled state were associated with a systemic metabolic phenotype. Pathway enrichment highlighted amino acid-related pathways as the dominant altered category, including amino acid biosynthesis and D-amino acid metabolism, which is consistent with previous epilepsy metabolomics studies (Eid, 2022; Rho and Boison, 2022; Lian et al., 2024; Hanin et al., 2024; Kang et al., 2025).

A key feature of our data is the convergence on amino acid metabolism. Multiple enriched pathways (including glycine/serine/threonine, histidine, arginine/proline, and phenylalanine-related metabolism) suggest coordinated remodeling rather than isolated metabolite fluctuation. This interpretation is biologically plausible because amino acid metabolism is tightly linked to neurotransmitter homeostasis and neuronal excitability (Perucca et al., 2023a; Olsen et al., 2024; Zhou et al., 2023; Xiong et al., 2024). Disturbance of excitatory-inhibitory balance, especially glutamatergic and GABAergic signaling, is a recognized mechanism in seizure generation and propagation (Perucca et al., 2023b; Olsen et al., 2024; Xiong et al., 2024; Bindila et al., 2022). The hippocampus-restricted analysis of the human TLE-HS transcriptomic dataset provided complementary pathway-level context for this interpretation, although it should not be regarded as direct validation of the plasma metabolomic findings (Galvis-Montes et al., 2023; Tröscher et al., 2023; Kishore et al., 2023).

In addition to amino acid pathways, central carbon and energy-related metabolism were also enriched. Given the high energetic demand during recurrent neuronal firing, changes in carbon flux and mitochondrial-associated metabolism are expected in epilepsy (Eid, 2022; Savitz, 2019; Ong et al., 2021). These findings support a model in which neurotransmitter precursor metabolism and bioenergetic stress are coupled during repeated seizure induction or the PTZ-kindled state (Savitz, 2019).

Previous metabolomic studies across experimental paradigms and clinical biospecimens have identified recurrent, although context-dependent, alterations in amino acid, energy, and lipid metabolism in seizure disorders. In a pilocarpine model, untargeted metabolomic profiling of hippocampal extracts collected at 3 h, 1 week, and 2 weeks after status epilepticus revealed stage-dependent alterations in amino acid- and lipid-related pathways, with tryptophan, N-acetylornithine, N-acetyl-L-aspartate, glutamine, adenosine, and cholesterol reported as putatively annotated differential metabolites (Meier et al., 2023). In an acute kainate-induced mouse seizure model, MALDI mass-spectrometry imaging demonstrated a massive reduction in ATP and a moderate reduction in ADP, particularly in the hippocampal CA3 region; complementary metabolomic data were interpreted as indicating accelerated glycolysis and possibly increased TCA-cycle activity in response to acute energy depletion (Sugiura et al., 2011). Clinical CSF studies have likewise identified metabolic abnormalities, although these appear to depend on age, clinical context, and treatment exposure. In pediatric patients, lower 2-ketoglutarate and higher pyridoxamine and tyrosine were observed specifically in children aged 0–5 years with epilepsy, whereas children aged 6–17 years showed lower 1,5-anhydroglucitol; valproate exposure was independently associated with several amino acid-related metabolites (Akiyama et al., 2020). In adults with status epilepticus, untargeted plasma and CSF metabolomics implicated amino acid metabolism, pyrimidine metabolism, and lipid homeostasis, with the tryptophan–kynurenine pathway showing the most significant alteration (Hanin et al., 2024). Plasma metabolomic and lipidomic profiling of valproate-treated children further showed lower fatty acid and glycerophospholipid levels but higher triglyceride levels in treatment nonresponders receiving polytherapy than in seizure-free responders receiving valproate monotherapy (Guo et al., 2023). Collectively, these studies point to recurrent involvement of amino acid metabolism, bioenergetic regulation, and lipid remodeling across different seizure-related conditions. However, variation in individual metabolites and their direction of change indicates that these findings should not be interpreted as a universal epilepsy metabolomic signature, because species, experimental model, disease or seizure stage, tissue or biofluid, age, treatment response, and antiseizure medication exposure may all influence the observed profiles.

LASSO feature selection identified six candidate metabolic features. Among them, ILA and a DG species were prioritized for biological interpretation because of their relevance to tryptophan-related and lipid-related metabolism. The other LASSO-selected features were reported in the Supplementary material with annotation information regarding their putative exogenous, pharmaceutical, dietary/environmental, or uncertain origins. Both metabolites were elevated in the epilepsy group. ILA is a tryptophan-derived indole metabolite linked to host-microbiota metabolic interactions (Cryan et al., 2019; Sorboni et al., 2022; Morais et al., 2020; Türay et al., 2023; Yang et al., 2024b), suggesting that altered tryptophan handling may contribute to the observed systemic phenotype. Nevertheless, the biological source of circulating ILA cannot be determined from the present data because gut microbial composition and microbial metabolic activity were not measured. Thus, the association between ILA and microbiota-related signaling should be considered hypothesis-generating. DG (20:3n6/0:0/20:3n6), a diacylglycerol species involved in lipid synthesis and intracellular signaling, may reflect altered lipid metabolism. However, an altered plasma DG feature does not by itself demonstrate changes in intracellular DG-mediated signaling (Avalos et al., 2025; Tröscher et al., 2023; Chen et al., 2025). Moreover, because the metabolite annotation was derived from untargeted metabolomics, confirmation using authentic standards and targeted quantitative analysis is required. Accordingly, this DG feature is best regarded as a candidate lipid-associated signature of the PTZ-kindled state.

The human hippocampal transcriptomic analysis was included to provide complementary biological context. KEGG over-representation analysis of differentially expressed genes identified pathway-level alterations relevant to neurotransmission, lipid metabolism, and inflammatory/stress responses. Representative genes mapping to the metabolic pathways identified in the mouse metabolomic analysis—including PHGDH, PSAT1, GAD1, FASN, and TDO2—showed differential expression between TLE-HS patients and controls. However, given the differences in species, tissue type, and study cohort, the metabolomic and transcriptomic findings should be interpreted as parallel observations rather than direct evidence of cross-species molecular coupling.

When considered together, the findings most consistently support alterations in amino acid metabolism in the PTZ-kindled state. The elevations of ILA and the putatively annotated DG feature additionally suggest possible involvement of tryptophan-associated and lipid-related metabolism, respectively, although these observations require targeted validation. The human hippocampal transcriptomic analysis provided complementary molecular context for epilepsy-related pathway alterations but did not directly validate the candidate plasma metabolites or their associated metabolic pathways (Yang W. et al., 2024).

This study has several limitations. First, metabolomics was performed in peripheral blood rather than brain tissue; therefore, central and peripheral metabolic changes cannot be directly equated. Second, gut microbiota composition was not profiled, so microbiota-related interpretations should be considered hypothesis-generating. Third, the sample size was modest, and independent validation (including targeted quantification and external cohorts) is still required. Fourth, spontaneous seizure activity was not monitored in this study, and the metabolic profile may reflect the kindled state rather than a fully developed chronic epilepsy phenotype. Additionally, because PTZ is a chemical convulsant, some metabolic alterations may reflect direct pharmacological effects of PTZ on hepatic or peripheral metabolism rather than seizure-related changes. The cross-sectional design of this study cannot distinguish between these possibilities. Future work integrating targeted metabolomics, microbiome sequencing, and functional validation will be essential to clarify causal pathways.

In summary, this study identified a distinct peripheral metabolic signature associated with PTZ-induced state, characterized primarily by alterations in amino acid-related pathways together with changes in tryptophan- and lipid-related metabolism. Indolelactic acid and DG (20:3n6/0:0/20:3n6) emerged as candidate plasma metabolites associated with the PTZ-kindled state, while reanalysis of a public human temporal lobe epilepsy transcriptomic dataset provided complementary human transcriptomic context for the observed metabolic alterations (Kang et al., 2025; Hanin et al., 2024; Lian et al., 2024). Therefore, these findings should be interpreted as hypothesis-generating rather than definitive. Nevertheless, the present work provides a systems-level framework for understanding epilepsy-associated metabolic remodeling and supports future studies aimed at targeted validation and mechanistic investigation.

## 5 Conclusion

This study suggests that PTZ-kindled state is accompanied by systemic metabolic alterations in peripheral blood. The most prominent alterations were concentrated in amino acid-associated pathways, with additional involvement of tryptophan-related and lipid metabolism. LASSO-based feature selection identified ILA and a DG species as candidate discriminative metabolites, both elevated in PTZ-kindled mice. When interpreted together with public human TLE transcriptomic evidence, these findings suggest the involvement of multiple metabolic domains spanning amino acid, tryptophan, and lipid axes. Although causal mechanisms require further validation, our results provide biologically coherent candidate signatures and a practical framework for future biomarker and mechanism-oriented studies.

## Data Availability

Publicly available datasets were analyzed in this study. This data can be found at: https://www.ncbi.nlm.nih.gov/geo/download/?acc=GSE256068.

## Glossary

AGC: automatic gain control
BMDB: BGI Metabolome Database
CON: control
DG: diacylglycerol
EDTA: ethylenediaminetetraacetic acid
FASN: fatty acid synthase
GABA: gamma-aminobutyric acid
GAD1: glutamate decarboxylase 1
GEO: Gene Expression Omnibus
HMDB: Human Metabolome Database
ILA: indolelactic acid
KEGG: Kyoto Encyclopedia of Genes and Genomes
LASSO: least absolute shrinkage and selection operator
LC–MS: liquid chromatography-mass spectrometry
OPLS-DA: orthogonal partial least squares discriminant analysis
PCA: principal component analysis
PHGDH: phosphoglycerate dehydrogenase
PLS-DA: partial least squares discriminant analysis
PQN: probabilistic quotient normalization
PSAT1: phosphoserine aminotransferase 1
PTZ: pentylenetetrazol
QC: quality control
QC-RLSC: quality control-based robust LOESS signal correction
TDO2: tryptophan 2,3-dioxygenase
TLE: temporal lobe epilepsy
TLE-HS: temporal lobe epilepsy with hippocampal sclerosis
UHPLC–MS: ultra-high-performance liquid chromatography-mass spectrometry
VIP: variable importance in projection
VST: variance-stabilizing transformation

## References

[ref1] Agus A. Planchais J. Sokol H. (2018). Gut microbiota regulation of tryptophan metabolism in health and disease. Cell Host Microbe 23, 716–724. doi: 10.1016/j.chom.2018.05.003, 29902437

[ref2] Akiyama T. Saigusa D. Hyodo Y. Umeda K. Saijo R. Koshiba S. . (2020). Metabolic profiling of the cerebrospinal fluid in Pediatric epilepsy. Acta Med. Okayama 74, 65–72. doi: 10.18926/AMO/57955, 32099251

[ref3] Avalos J. C. Pellizza L. Aran M. (2025). Metabolic alterations associated with epileptic seizures detected by NMR spectroscopy. Sci. Rep. 15:29119. doi: 10.1038/s41598-025-14718-1, 40781139 PMC12334711

[ref4] Bian X. Shao X. (2024). Advances in the study of gut microbes in pediatric epilepsy. Epilepsy Behav 157:109899. doi: 10.1016/j.yebeh.2024.109899, 38885595

[ref5] Bindila L. Eid T. Mills J. D. Hildebrand M. S. Brennan G. P. Masino S. A. . (2022). A companion to the preclinical common data elements for proteomics, lipidomics, and metabolomics data in rodent epilepsy models. A report of the TASK3-WG4 omics working group of the ILAE/AES joint translational TASK force. Epilepsia Open 10, S206–S237. doi: 10.1002/epi4.12662, 36259125 PMC12375985

[ref6] Cerdán-Centeno K. T. González-Soto A. Y. Lares-López V. Sánchez-Vázquez L. D. Tapia-Estrada H. R. Túnez I. . (2026). Kynurenine pathway, Nrf2 and NF-κB cross-regulation in the CNS: an overview. Mol. Neurobiol. 63:527. doi: 10.1007/s12035-026-05802-2, 41894093 PMC13031210

[ref7] Chen Y. Nie Q. Song T. Zou X. Li Q. Zhang P. (2025). Integrated proteomics and Lipidomics analysis of Hippocampus to reveal the metabolic landscape of epilepsy. ACS Omega 10, 9351–9367. doi: 10.1021/acsomega.4c10085, 40092809 PMC11904687

[ref8] Chen W. Zhang P. Zhang X. Xiao T. Zeng J. Guo K. . (2024). Machine learning-causal inference based on multi-omics data reveals the association of altered gut bacteria and bile acid metabolism with neonatal jaundice. Gut Microbes 16:2388805. doi: 10.1080/19490976.2024.2388805, 39166704 PMC11340767

[ref9] Cryan J. F. O'Riordan K. J. Sandhu K. Peterson V. Dinan T. G. (2019). The gut microbiome in neurological disorders. Lancet. Neurol. 19, 179–194. doi: 10.1016/S1474-4422(19)30356-4, 31753762

[ref10] Ding M. Lang Y. Shu H. Shao J. Cui L. (2021). Microbiota-gut-brain axis and epilepsy: a review on mechanisms and potential therapeutics. Front. Immunol. 12:742449. doi: 10.3389/fimmu.2021.742449, 34707612 PMC8542678

[ref11] Eid T. (2022). Harnessing metabolomics to advance epilepsy research. Epilepsy Curr. 22, 123–129. doi: 10.1177/15357597221074518, 35444500 PMC8988727

[ref12] El-Sayed R. M. Fawzy M. N. Zaki H. F. Abd El-Haleim E. A. (2023). Neuroprotection impact of biochanin a against pentylenetetrazol-kindled mice: targeting NLRP3 inflammasome/TXNIP pathway and autophagy modulation. Int. Immunopharmacol. 115:109711. doi: 10.1016/j.intimp.2023.109711, 36640710

[ref13] François L. Romagnolo A. Luinenburg M. J. Anink J. J. Godard P. Rajman M. . (2024). Identification of gene regulatory networks affected across drug-resistant epilepsies. Nat. Commun. 15:2180. doi: 10.1038/s41467-024-46592-2, 38467626 PMC10928184

[ref14] Galvis-Montes D. S. van Loo K. M. J. van Waardenberg A. J. Surges R. Schoch S. Becker A. J. . (2023). Highly dynamic inflammatory and excitability transcriptional profiles in hippocampal CA1 following status epilepticus. Sci. Rep. 13:22187. doi: 10.1038/s41598-023-49310-y, 38092829 PMC10719343

[ref15] Guo H.-L. Wang W.-J. Dong N. Zhao Y.-T. Dai H.-R. Hu Y.-H. . (2023). Integrating metabolomics and lipidomics revealed a decrease in plasma fatty acids but an increase in triglycerides in children with drug-refractory epilepsy. Epilepsia Open 8, 466–478. doi: 10.1002/epi4.12712, 36808532 PMC10235554

[ref16] Hanin A. Chollet C. Demeret S. Di Meglio L. Castelli F. Navarro V. (2024). Metabolomic changes in adults with status epilepticus: a human case-control study. Epilepsia 65, 929–943. doi: 10.1111/epi.17899, 38339978

[ref17] Hughes A. Vangeenderhuysen P. De Graeve M. Pomian B. Nawrot T. S. Raes J. . (2025). Toward automated preprocessing of untargeted LC-MS-based metabolomics feature lists from human biofluids. Anal. Chem. 97, 122–129. doi: 10.1021/acs.analchem.4c03124, 39757901

[ref18] Kang W. Han Q. Chen M. Xue L. Zhao Z. Liu R. . (2025). Alterations in serum metabolomics predict drug-resistant epilepsy. Sci. Rep. 16:1003. doi: 10.1038/s41598-025-30741-8, 41339472 PMC12783295

[ref19] Kishore M. Pradeep M. Narne P. Jayalakshmi S. Panigrahi M. Patil A. . (2023). Regulation of Keap1-Nrf2 axis in temporal lobe epilepsy-hippocampal sclerosis patients may limit the seizure outcomes. Neurol. Sci. 44, 4441–4450. doi: 10.1007/s10072-023-06936-0, 37432566

[ref20] Krishnamurthy K. B. (2025). Epilepsy. Ann. Intern. Med. 178, ITC49–ITC64. doi: 10.7326/ANNALS-25-00494, 40194289

[ref21] Leach D. T. Stratton K. G. Irvahn J. Richardson R. Webb-Robertson B.-J. M. Bramer L. M. (2023). malbacR: a package for standardized implementation of batch correction methods for omics data. Anal. Chem. 95, 12195–12199. doi: 10.1021/acs.analchem.3c01289, 37551970

[ref22] Li F. You D. Li Y. Wang X. Lin Z. Shi X. . (2025). Multi-omics integration reveals gut microbiota dysbiosis and metabolic alterations of cerebrospinal fluid in children with epilepsy. Front. Microbiol. 16:1630062. doi: 10.3389/fmicb.2025.1630062, 41019533 PMC12461256

[ref23] Lian X. Liu Z. Liu S. Jin L. Wu T. Chen Y. . (2024). Alterations in serum metabolomics during the first seizure and after effective control of epilepsy. Sci. Rep. 14:19180. doi: 10.1038/s41598-024-68966-8, 39160238 PMC11333619

[ref24] Löscher W. Klein P. (2021). The pharmacology and clinical efficacy of antiseizure medications: from bromide salts to Cenobamate and beyond. CNS Drugs 35, 935–963. doi: 10.1007/s40263-021-00827-8, 34145528 PMC8408078

[ref25] Meier L. Bruginski E. Marafiga J. R. Caus L. B. Pasquetti M. V. Calcagnotto M. E. . (2023). Hippocampal metabolic profile during epileptogenesis in the pilocarpine model of epilepsy. Biomed. Chromatogr. 38:e5820. doi: 10.1002/bmc.5820, 38154955

[ref26] Morais L. H. Schreiber H. L. Mazmanian S. K. (2020). The gut microbiota-brain axis in behaviour and brain disorders. Nat. Rev. Microbiol. 19, 241–255. doi: 10.1038/s41579-020-00460-0, 33093662

[ref27] Olasunkanmi O. I. Zheng L. Zheng P. (2026). Gut-brain axis in health and brain disease. Chin. Med. J. 139, 799–827. doi: 10.1097/CM9.0000000000003920, 41630697 PMC12999160

[ref28] Olsen R. W. Wallner M. Rogawski M. A. (2024). “GABAA Receptors, Seizures, and Epilepsy,” in Jasper’s Basic Mechanisms of the Epilepsies, eds. J. L. Noebels, M. Avoli, M. A. Rogawski, A. Vezzani and A. V. Delgado-Escueta, 5th ed. New York, NY: Oxford University Press, 1025–1046. doi: 10.1093/med/9780197549469.003.0048

[ref29] Ong C. Damisah E. C. Gruenbaum S. E. Dhaher R. Deng Y. Sandhu M. R. S. . (2021). Increased branched-chain amino acids at baseline and hours before a spontaneous seizure in the human epileptic brain. Epilepsia 62, e88–e97. doi: 10.1111/epi.16920, 33949690 PMC11259155

[ref30] Perucca E. Bialer M. White H. S. (2023a). New GABA-targeting therapies for the treatment of seizures and epilepsy: I. Role of GABA as a modulator of seizure activity and recently approved medications acting on the GABA system. CNS Drugs 37, 755–779. doi: 10.1007/s40263-023-01027-2, 37603262 PMC10501955

[ref31] Perucca E. White H. S. Bialer M. (2023b). New GABA-targeting therapies for the treatment of seizures and epilepsy: II. Treatments in clinical development. CNS Drugs 37, 781–795. doi: 10.1007/s40263-023-01025-4, 37603261 PMC10501930

[ref32] Rho J. M. Boison D. (2022). The metabolic basis of epilepsy. Nat. Rev. Neurol. 18, 333–347. doi: 10.1038/s41582-022-00651-8, 35361967 PMC10259193

[ref33] Savitz J. (2019). The kynurenine pathway: a finger in every pie. Mol. Psychiatry 25, 131–147. doi: 10.1038/s41380-019-0414-4, 30980044 PMC6790159

[ref34] Shaw C. Hess M. Weimer B. C. (2023). Microbial-derived tryptophan metabolites and their role in neurological disease: anthranilic acid and anthranilic acid derivatives. Microorganisms 11:1825. doi: 10.3390/microorganisms11071825, 37512997 PMC10384668

[ref35] Sorboni S. G. Moghaddam H. S. Jafarzadeh-Esfehani R. Soleimanpour S. (2022). A comprehensive review on the role of the gut microbiome in human neurological disorders. Clin. Microbiol. Rev. 35:e0033820. doi: 10.1128/CMR.00338-20, 34985325 PMC8729913

[ref36] Sugiura Y. Taguchi R. Setou M. (2011). Visualization of spatiotemporal energy dynamics of hippocampal neurons by mass spectrometry during a kainate-induced seizure. PLoS One 6:e17952. doi: 10.1371/journal.pone.0017952, 21445350 PMC3062556

[ref37] Tröscher A. R. Mair K. M. Verdú de Juan L. Köck U. Steinmaurer A. Baier H. . (2023). Temporal lobe epilepsy with GAD antibodies: neurons killed by T cells not by complement membrane attack complex. Brain 146, 1436–1452. doi: 10.1093/brain/awac404, 36314080 PMC10115353

[ref38] Tseng C. Y. Salguero J. A. Breidenbach J. D. Solomon E. Sanders C. K. Harvey T. . (2025). Evaluation of normalization strategies for mass spectrometry-based multi-omics datasets. Metabolomics 21:98. doi: 10.1007/s11306-025-02297-1, 40593232 PMC12214035

[ref39] Türay S. Cangür Ş. Kahraman G. Kayabaşı E. Çetiner Ö. F. Aydın B. . (2023). Can the gut microbiota serve as a guide to the diagnosis and treatment of childhood epilepsy? Pediatr. Neurol. 145, 11–21. doi: 10.1016/j.pediatrneurol.2023.04.006, 37245274

[ref40] Wang J. Wang Y. Zeng Y. Huang D. (2022). Feature selection approaches identify potential plasma metabolites in postmenopausal osteoporosis patients. Metabolomics 18:86. doi: 10.1007/s11306-022-01937-0, 36318345

[ref41] Xia H.-H. Huang J.-Z. (2026). Tryptophan metabolism at the crossroads of the neuro-immuno-microbial axis: implications for precision medicine in chronic diseases. Front. Cell. Infect. Microbiol. 15:1707850. doi: 10.3389/fcimb.2025.1707850, 41602107 PMC12833013

[ref42] Xiong W. Yeo T. May J. T. M. Demmers T. Ceronie B. Ramesh A. . (2024). Distinct plasma metabolomic signatures differentiate autoimmune encephalitis from drug-resistant epilepsy. Ann. Clin. Trans. Neurol. 11, 1897–1908. doi: 10.1002/acn3.52112, 39012808 PMC11251473

[ref43] Yan R. Zhang L. Chen Y. Zheng Y. Xu P. Xu Z. (2025). Therapeutic potential of gut microbiota modulation in epilepsy: a focus on short-chain fatty acids. Neurobiol. Dis. 209:106880. doi: 10.1016/j.nbd.2025.106880, 40118219

[ref44] Yang J. Cai J.-H. Wu T.-X. Gao Z.-Q. Zhou C. Wu Q. . (2024). Salvinorin a ameliorates pilocarpine-induced seizures by regulating hippocampal microglia polarization. J. Ethnopharmacol. 335:118697. doi: 10.1016/j.jep.2024.118697, 39154669

[ref45] Yang W. Cui H. Wang C. Wang X. Yan C. Cheng W. (2024). A review of the pathogenesis of epilepsy based on the microbiota-gut-brain-axis theory. Front. Mol. Neurosci. 17:1454780. doi: 10.3389/fnmol.2024.1454780, 39421261 PMC11484502

[ref46] Zhou K. Jia L. Mao Z. Si P. Sun C. Qu Z. . (2023). Integrated macrogenomics and metabolomics explore alterations and correlation between gut microbiota and serum metabolites in adult epileptic patients: a pilot study. Microorganisms 11:2628. doi: 10.3390/microorganisms11112628, 38004640 PMC10672912

