# Supplementary material for: Amino acid- and lipid-related metabolic remodeling in PTZ-kindled mice reveals candidate plasma signatures of chronic epilepsy
Source: Front Neurosci. 2026 Aug 20;20:1889410. doi: 10.3389/fnins.2026.1889410 (PMC13538445; doi:10.3389/fnins.2026.1889410)
Supplement: Supplementary file 1 [file Data_Sheet_1.PDF]

Supplementary Materials

Supplementary Table S1. Individual seizure phenotype data for PTZ-treated mice.

Mouse ID	Injection No.	Racine Score	Latency (s)	Duration (s)
PTZ-1	1	NR	NA	NA
PTZ-1	2	2	NA	NA
PTZ-1	3	3	NA	NA
PTZ-1	4	3	NA	NA
PTZ-1	5	3	NA	NA
PTZ-1	6	3	691.8	18.3
PTZ-1	7	5	612.8	31.9
PTZ-1	8	5	381.2	27.2
PTZ-1	9	5	402.6	23.9
PTZ-1	10	5	396.2	19.3
PTZ-1	11	5	332.1	19.2
PTZ-2	1	NR	NA	NA
PTZ-2	2	NR	NA	NA
PTZ-2	3	NR	NA	NA
PTZ-2	4	1	NA	NA
PTZ-2	5	1	NA	NA
PTZ-2	6	2	NA	NA
PTZ-2	7	3	NA	NA
PTZ-2	8	3	NA	NA
PTZ-2	9	5	655.8	22
PTZ-2	10	5	279.5	18.4
PTZ-2	11	5	188.3	18.1
PTZ-3	1	NR	NA	NA
PTZ-3	2	NR	NA	NA
PTZ-3	3	1	NA	NA
PTZ-3	4	2	NA	NA
PTZ-3	5	2	NA	NA
PTZ-3	6	3	NA	NA
PTZ-3	7	3	NA	NA
PTZ-3	8	5	253.4	18.9
PTZ-3	9	5	101	23.2
PTZ-3	10	5	186.3	22.6
PTZ-3	11	5	118.6	24.8
PTZ-4	1	NR	NA	NA
PTZ-4	2	2	NA	NA
PTZ-4	3	2	NA	NA
PTZ-4	4	2	NA	NA
PTZ-4	5	3	NA	NA
PTZ-4	6	3	NA	NA

PTZ-4	7	4	281.5	18.2
PTZ-4	8	5	216.7	15.2
PTZ-4	9	5	293.3	25.7
PTZ-4	10	5	155.2	24
PTZ-4	11	5	539.5	49.1
PTZ-5	1	NR	NA	NA
PTZ-5	2	NR	NA	NA
PTZ-5	3	NR	NA	NA
PTZ-5	4	1	NA	NA
PTZ-5	5	1	NA	NA
PTZ-5	6	2	NA	NA
PTZ-5	7	2	NA	NA
PTZ-5	8	3	NA	NA
PTZ-5	9	5	164.2	23.6
PTZ-5	10	5	158.1	18.5
PTZ-5	11	5	167.6	24.2
PTZ-6	1	NR	NA	NA
PTZ-6	2	NR	NA	NA
PTZ-6	3	NR	NA	NA
PTZ-6	4	1	NA	NA
PTZ-6	5	2	NA	NA
PTZ-6	6	2	NA	NA
PTZ-6	7	3	NA	NA
PTZ-6	8	3	NA	NA
PTZ-6	9	5	291.5	31.6
PTZ-6	10	5	548.2	20.9
PTZ-6	11	5	249.3	46.3
PTZ-7	1	NR	NA	NA
PTZ-7	2	NR	NA	NA
PTZ-7	3	2	NA	NA
PTZ-7	4	3	NA	NA
PTZ-7	5	3	NA	NA
PTZ-7	6	5	101.8	25.4
PTZ-7	7	5	991.8	25
PTZ-7	8	5	119.5	18.2
PTZ-7	9	6	158.5	55.2
PTZ-8	1	NR	NA	NA
PTZ-8	2	NR	NA	NA
PTZ-8	3	NR	NA	NA
PTZ-8	4	NR	NA	NA
PTZ-8	5	NR	NA	NA
PTZ-8	6	NR	NA	NA
PTZ-8	7	1	NA	NA
PTZ-8	8	1	NA	NA

PTZ-8	9	2	NA	NA
PTZ-8	10	3	NA	NA
PTZ-8	11	3	NA	NA

Mice received intraperitoneal injections of PTZ (35 mg/kg) every other day for a total of 11 injections. Seizure severity was evaluated using the Racine scale: I, facial automatisms and rhythmic head nodding; II, head clonus with neck muscle involvement; III, unilateral forelimb clonus; IV, bilateral forelimb clonus with rearing; V, rearing and falling accompanied by generalized tonic-clonic convulsions; VI, loss of posture with continuous jumping, wild running, or death. NR indicates no observable seizure response. Latency was defined as the time from completion of PTZ injection to the onset of the first Racine stage IV or higher seizure. Duration was defined as the time from onset of Racine stage IV or higher seizure behavior to recovery of normal posture and spontaneous locomotion. Latency and duration were recorded only when generalized seizures (Racine stage IV or higher) occurred; otherwise, NA (not applicable) was assigned. Successful kindling was defined as Racine stage IV or higher seizures for at least three consecutive stimulations. Of the eight PTZ-treated mice, six (PTZ-1 through PTZ-6) met the kindling criterion and were included in the metabolomic analysis. PTZ-7 met the kindling criterion at injection 6 but died during seizure activity at injection 9 (Racine stage VI) and was therefore excluded from metabolomic analysis. PTZ-8 failed to meet the kindling criterion (maximum Racine score = 3) and was excluded. Control mice (CON-1 through CON-6) received saline and showed no seizure behavior (data not shown). NR, no seizure response; s, seconds; NA, not applicable.

Supplementary Table S2. Variance captured by PCA, PLS-DA, and OPLS-DA models.

(A) PCA

Component	R^2X (%)	Cumulative R^2X (%)
PC1	26.83	26.83
PC2	15.47	42.29
PC3	10.06	52.35
PC4	9.07	61.41
PC5	7.73	69.14
PC6	6.27	75.41

(B) PLS-DA

Component	R^2X (%)	R^2X(cum) (%)	R^2Y (%)	R^2Y(cum) (%)
Comp1	22.43	22.43	77.09	77.09
Comp2	15.52	37.95	18.74	95.83
Comp3	11.00	48.95	3.91	99.74
Comp4	9.04	58.00	0.24	99.98
Comp5	5.90	63.90	0.02	100.00

(C) OPLS-DA

Component	Type	R^2X (%)	R^2X(cum) (%)
Predictive 1	Predictive	22.43	22.43
Orthogonal 1	Orthogonal	18.69	41.12
Orthogonal 2	Orthogonal	10.62	51.75
Orthogonal 3	Orthogonal	9.07	60.81

Variance explained by PCA, PLS-DA, and OPLS-DA models for plasma metabolite profiling of PTZ-kindled mice versus controls (n = 6 per group). Metabolite data were $\log_2$-transformed and Pareto-scaled prior to multivariate analysis. For PCA, R^2X represents the proportion of variance explained by each principal component (PC1–PC6). For PLS-DA, R^2X and R^2Y represent the proportion of variance in X (metabolites) and Y (group classification) explained by each component (Comp1–Comp5), respectively. For OPLS-DA, R^2X values are shown for the predictive component (Predictive 1) and orthogonal components (Orthogonal 1–3). Abbreviations: PCA, principal component analysis; PLS-DA, partial least squares discriminant analysis; OPLS-DA, orthogonal partial least squares discriminant analysis; PTZ, pentylenetetrazole; CON, control; Cum, cumulative.

Supplementary Table S3. Significantly altered plasma metabolites in PTZ-kindled mice (PTZ vs CON).

No.	Metabolite ID	Name	HMDB ID	Log_2FC(PTZ-vs-CON)	VIP(PTZ-vs-CON)	*P*value(PTZ-vs-CON)	Qvalue(PTZ-vs-CON)	Ratio(PTZ-vs-CON)	Regulation
1	2.812_155.0927219	Tryptoline	HMDB0012488	5.241	4.281	0.0196	0.3187	37.806	Up
2	0.078_692.0180326	DG(20:3n6/0:0/20:3n6)	HMDB0056321	3.815	4.857	0.0000	0.0021	14.079	Up
3	5.658_245.1365338	Pyrogallol-2-O-sulphate	HMDB0060018	-2.87	3.833	0.0016	0.2288	0.137	Down
4	1.129_147.0289386	Pentafluoropropionic acid	HMDB0256333	-1.751	2.432	0.0338	0.3550	0.297	Down
5	6.703_654.4326680	Ranimustine	HMDB0257109	-1.628	2.809	0.0164	0.3089	0.324	Down
6	6.485_391.2867840	3,3'-Bisjuglone	HMDB0033936	-1.582	1.928	0.0440	0.3874	0.334	Down
7	6.467_463.2736736	Epidepride	HMDB0251845	-1.517	2.497	0.0244	0.3368	0.349	Down
8	3.963_337.0391209	Myricetin	HMDB0002755	1.507	2.745	0.0246	0.3373	2.842	Up
9	2.420_294.1016421	Lofexidine	HMDB0015606	1.5	2.831	0.0081	0.2536	2.829	Up
10	6.056_197.0812022	Phenyl dihydrogen phosphate	HMDB0256445	-1.462	2.76	0.0097	0.2650	0.363	Down
11	7.375_305.2487598	8Z,11Z,14Z-Eicosatrienoic acid	HMDB0002925	-1.438	2.445	0.0328	0.3532	0.369	Down

12	4.133_144.0914481	7-Nitroindazole	HMDB0247275	1.427	2.788	0.0003	0.2062	2.688	Up
13	7.353_567.4641892	Sesaminol glucoside	HMDB0041209	-1.411	2.173	0.0377	0.3677	0.376	Down
14	5.972_455.3150027	Trovafloxacin	HMDB0014823	-1.382	2.452	0.0044	0.2376	0.384	Down
15	4.139_321.0440166	Quercetin	HMDB0005794	1.37	2.813	0.0013	0.2288	2.584	Up
16	7.510_331.2645877	(+/-)-CP 47,497-C8-Homolog		-1.363	2.169	0.0366	0.3635	0.389	Down
17	5.230_338.1223905	Chlorzoxazone	HMDB0014500	-1.349	2.73	0.0075	0.2536	0.393	Down
18	5.049_335.0596714	Perfluorobutanesulfonic acid	HMDB0246220	1.347	2.797	0.0055	0.2403	2.544	Up
19	6.855_579.4275123	Maclurin 3-C-(6''-p-hydroxybenzoyl-glucoside)	HMDB0032646	-1.341	2.381	0.0480	0.3953	0.395	Down
20	2.798_188.0353130	alpha-Cyano-3-hydroxycinnamic acid		1.335	2.689	0.0004	0.2062	2.523	Up
21	3.667_237.1244486	Trimellitic anhydride	HMDB0259233	1.289	1.986	0.0396	0.3736	2.443	Up
22	2.901_197.1547451	4-Hydrazinylpyridine-3-carboxamide	HMDB0258083	1.21	2.571	0.0032	0.2288	2.314	Up

23	3.121_206.0449618	5,7-Dichloropyrazolo[1,5-a]pyrimidine	HMDB0246869	1.209	2.186	0.0034	0.2288	2.311	Up
24	3.865_226.1165258	6-Formylpterin	HMDB0247066	1.182	2.197	0.0255	0.3405	2.269	Up
25	4.139_144.0454938	4-Indolecarbaldehyde		1.181	2.609	0.0005	0.2062	2.267	Up
26	7.306_267.2328884	Isopropyl beta-D-glucoside	HMDB0032705	-1.168	2.128	0.0343	0.3563	0.445	Down
27	3.972_229.1342236	7-Hydroxy-4-(trifluoromethyl)coumarin	HMDB0247257	-1.16	2.106	0.0154	0.3016	0.447	Down
28	6.776_465.3230268	Urolithin C 3-glucuronide	HMDB0142405	-1.154	2.094	0.0311	0.3524	0.449	Down
29	7.623_375.2520334	Salvianolic acid D	HMDB0041773	-1.152	2.109	0.0262	0.3432	0.45	Down
30	4.139_145.0488110	1,3,2lambda~6~-Dioxathietane-2,2-dione	HMDB0246970	1.139	2.582	0.0028	0.2288	2.202	Up
31	2.858_349.1506980	Glycerylphosphorylinositol	HMDB0252859	1.112	2.38	0.0056	0.2403	2.162	Up
32	7.480_563.5065087	Rubratoxin B	HMDB0257358	-1.089	2.236	0.0178	0.3148	0.47	Down
33	7.636_307.2644077	Cannithrene 2	HMDB0301762	-1.089	2.016	0.0328	0.3532	0.47	Down

34	5.853_323.1832924	Aureol	HMDB0037921	-1.088	2.255	0.0074	0.2536	0.47	Down
35	6.496_462.3311082	Mometasone	HMDB0014902	-1.073	2.392	0.0009	0.2218	0.475	Down
36	7.387_602.5266936	Azidopine	HMDB0248800	-1.054	2.116	0.0311	0.3524	0.482	Down
37	0.687_496.9431085	Sporidesmin	HMDB0258439	-1.054	2.434	0.0127	0.2820	0.482	Down
38	6.472_451.3069131	Iophendylate	HMDB0015318	-1.051	2.153	0.0183	0.3155	0.482	Down
39	0.679_219.0101971	Phosphohydroxypyruvic acid	HMDB0001024	1.051	2.726	0.0189	0.3168	2.072	Up
40	6.824_457.2364846	Oleoyl-L-alpha-lysophosphatidic acid	HMDB0007855	-1.048	2.076	0.0124	0.2792	0.484	Down
41	2.721_137.0244030	3-Hydroxybenzoic acid	HMDB0002466	1.038	2.11	0.0282	0.3460	2.054	Up
42	3.331_213.1132784	Arkofix	HMDB0248602	1.03	2.139	0.0276	0.3437	2.042	Up
43	0.708_261.0109624	Iron vitriol	HMDB0303498	-1.025	1.918	0.0448	0.3892	0.492	Down
44	4.466_320.1538321	N5-Carboxyaminoimidazole ribonucleotide	HMDB0012268	-1.004	2.343	0.0298	0.3496	0.498	Down
45	3.353_169.1234715	1,2,3-Oxadiazol-5-amine	HMDB0258647	0.992	2.247	0.0019	0.2288	1.989	Up

46	3.594_245.1505267	Mannoheptulose	HMDB0254327	0.987	2.24	0.0078	0.2536	1.983	Up
47	5.654_356.2429756	Imidazoleacetic acid ribotide	HMDB0006032	0.977	2.233	0.0245	0.3373	1.968	Up
48	3.942_345.1194819	3-oxobrimonidine	HMDB0061102	0.974	2.245	0.0010	0.2280	1.964	Up
49	2.888_301.0878491	Irsogladine	HMDB0253587	0.972	2.387	0.0006	0.2062	1.962	Up
50	5.008_262.1446730	7-(Trifluoromethoxy)indolin-2-one	HMDB0247224	0.965	1.725	0.0497	0.3978	1.952	Up
51	2.896_302.0841282	Piridronic acid	HMDB0256596	0.961	2.445	0.0000	0.1114	1.947	Up
52	1.326_215.0925055	2,2,2-Trifluoro-N-(2-nitrophenyl)acetamide	HMDB0247856	0.952	2.33	0.0079	0.2536	1.935	Up
53	0.717_448.8402866	Cyhalothrin	HMDB0250689	-0.935	2.396	0.0035	0.2298	0.523	Down
54	7.492_282.2519617	Citrusinine I	HMDB0030374	-0.934	2.058	0.0194	0.3178	0.524	Down
55	4.058_146.0600143	2-Hydroxyquinoline		0.932	2.235	0.0006	0.2062	1.907	Up
56	5.814_242.1211254	3-Azido-7-hydroxycoumarin	HMDB0245825	-0.932	1.814	0.0391	0.3721	0.524	Down

57	0.714_590.75 77900	Calcium bromate	HMDB0303630	-0.93	2.172	0.0118	0.2782	0.525	Down
58	8.745_818.58 94065	Cyanidin 3-O-dimalonyl-laminaribioside	HMDB0029242	-0.919	2.061	0.0326	0.3532	0.529	Down
59	2.391_189.98 96917	3-Aminosalicylic acid	HMDB0001972	0.915	1.593	0.0334	0.3546	1.885	Up
60	2.391_188.98 63366	Hydroquinone sulfate	HMDB0240263	0.912	1.605	0.0308	0.3520	1.882	Up
61	5.097_186.12 16378	2-Aminomuconic acid semialdehyde	HMDB0001280	0.912	2.058	0.0241	0.3368	1.882	Up
62	7.730_395.34 96988	Sivifene	HMDB0258321	0.894	1.779	0.0179	0.3148	1.858	Up
63	7.419_573.45 32809	Clitocine	HMDB0033718	-0.888	2.047	0.0201	0.3218	0.54	Down
64	0.669_428.94 80879	Clomifene	HMDB0015020	0.887	2.212	0.0389	0.3720	1.849	Up
65	7.492_281.24 86017	Elaidic acid	HMDB0000573	-0.887	1.986	0.0234	0.3345	0.541	Down
66	0.858_150.05 83983	Methionine	HMDB0033951	0.875	2.139	0.0051	0.2403	1.834	Up
67	6.065_291.19 51305	6-Hydroxy-5-fluorocytosine	HMDB0247069	-0.875	2.142	0.0151	0.2997	0.545	Down
68	8.154_899.62 55635	Dinofenate	HMDB0251423	-0.868	1.785	0.0427	0.3846	0.548	Down

69	2.505_400.1728785	Mercury	HMDB0003625	0.867	2.01	0.0304	0.3514	1.824	Up
70	3.981_227.1289220	Traumatic acid	HMDB0000933	0.867	1.957	0.0342	0.3563	1.824	Up
71	5.035_585.2705552	Bilirubin	HMDB0000054	-0.866	1.554	0.0322	0.3532	0.548	Down
72	5.100_185.1183212	10-HDA		0.866	2.009	0.0194	0.3178	1.823	Up
73	3.901_199.0070723	4-Vinylphenol sulfate	HMDB0062775	0.857	1.594	0.0310	0.3524	1.811	Up
74	4.744_255.1601479	Cyclopentylacetic acid		0.856	2.313	0.0008	0.2106	1.81	Up
75	2.896_204.0665908	Indolelactic acid	HMDB0000671	0.853	2.242	0.0000	0.0180	1.806	Up
76	4.570_167.0555333	Ammelide	HMDB0248340	0.845	1.891	0.0366	0.3639	1.796	Up
77	6.332_265.1949756	Benzoic anhydride	HMDB0249022	0.843	2.072	0.0217	0.3306	1.794	Up
78	3.039_298.0965299	5'-S-Methyl-5'-thioadenosine	HMDB0001173	-0.843	1.809	0.0362	0.3617	0.558	Down
79	8.800_146.1653089	3-Aminopiperidine-2,6-dione	HMDB0242134	0.84	2.188	0.0081	0.2536	1.79	Up
80	6.055_221.1173784	Triethyl phosphate	HMDB0259191	-0.839	1.987	0.0235	0.3354	0.559	Down
81	0.677_177.1233373	Sulfonylthiourea	HMDB0258599	0.825	2.198	0.0083	0.2541	1.771	Up

82	0.646_480.9620134	Dorzagliatin	HMDB0251591	0.821	2.212	0.0500	0.3978	1.767	Up
83	0.652_210.8423803	2,2,2-Trichloroethyl chloroformate	HMDB0245336	-0.818	2.116	0.0182	0.3155	0.567	Down
84	8.668_801.0393769	PE(22:5(4Z,7Z,10Z,13Z,19Z)-O(16,17)/DiMe(9,3))	HMDB0285049	-0.807	1.832	0.0035	0.2298	0.571	Down
85	0.686_282.8235538	Praseodymium	HMDB0303931	-0.794	1.969	0.0213	0.3290	0.577	Down
86	4.856_181.9729559	Isopropyldichloromethyl ether	HMDB0253680	-0.793	1.935	0.0311	0.3524	0.577	Down
87	2.601_188.0928713	1-hydroxyhexanoylglycine	HMDB0094718	0.789	2.143	0.0095	0.2628	1.728	Up
88	0.556_147.1685809	4-Fluorocyclohexane-1-carboxylic acid	HMDB0246426	0.788	2.185	0.0048	0.2403	1.727	Up
89	0.740_160.1333333	Acetyl-beta-methylcholine	HMDB0015654	-0.778	1.793	0.0475	0.3949	0.583	Down
90	0.858_151.0617498	Pentafluorobenzene	HMDB0256258	0.778	2.053	0.0004	0.2062	1.715	Up
91	0.727_668.7758238	Hydroxyhomodestruxin B	HMDB0039897	-0.776	2.222	0.0006	0.2062	0.584	Down
92	6.035_267.1952906	1,4-Diacetoxy-2,3-dicyanobenzene	HMDB0244205	0.775	1.951	0.0158	0.3035	1.712	Up

93	0.858_133.0318095	2-Hydroxy-4-(methylthio)butanoic acid	HMDB0037115	0.763	1.85	0.0208	0.3260	1.697	Up
94	6.218_359.2806370	4'-Hydroxy Nimesulide	HMDB0246630	-0.761	1.676	0.0393	0.3727	0.59	Down
95	7.418_357.3839627	Phenacemide	HMDB0015253	0.755	1.646	0.0196	0.3187	1.688	Up
96	0.555_129.1386127	2-dehydropantoate	HMDB0304068	0.75	2.145	0.0064	0.2457	1.681	Up
97	0.728_604.8724557	Cer(d16:1/TXB2)	HMDB0289856	-0.734	2.113	0.0049	0.2403	0.601	Down
98	8.253_332.0483484	Carboxyifosfamide	HMDB0060692	0.731	1.639	0.0233	0.3345	1.66	Up
99	4.285_263.0593218	Carnosine	HMDB0000033	0.731	1.758	0.0188	0.3168	1.659	Up
100	0.980_366.1092293	Dehydrochloramphenicol	HMDB0250945	-0.728	2.045	0.0030	0.2288	0.604	Down
101	0.556_146.1652541	Spermidine	HMDB0001257	0.728	2.092	0.0081	0.2536	1.656	Up
102	8.008_914.5911501	(2E)-Glutaconylcarnitin-CoA	HMDB0301422	-0.727	1.892	0.0126	0.2808	0.604	Down
103	3.501_236.0927748	phenylacetylthreonine	HMDB0341374	0.721	1.972	0.0109	0.2741	1.648	Up
104	3.179_243.1697635	Aspartyl-Serine	HMDB0028762	-0.719	1.734	0.0193	0.3178	0.608	Down

105	6.850_541.8310646	DG(18:4(6Z,9Z,12Z,15Z)/14:1(9Z)/0:0)	HMDB0007328	-0.707	1.929	0.0081	0.2536	0.613	Down
106	3.655_540.1675809	Deltamethrin	HMDB0041866	-0.704	1.712	0.0385	0.3701	0.614	Down
107	0.603_389.0509929	FG-4592		-0.7	2.092	0.0039	0.2298	0.615	Down
108	5.253_648.2455678	Tricosane	HMDB0061866	-0.698	1.705	0.0375	0.3669	0.616	Down
109	1.713_217.1080796	2,3,5,6-Tetrafluorobenzenethiol	HMDB0245438	0.69	1.622	0.0412	0.3807	1.613	Up
110	8.097_588.5473255	Tripeptide	HMDB0259261	-0.684	1.57	0.0410	0.3798	0.622	Down
111	0.723_670.7735962	11-trans-Leukotriene C4	HMDB0005095	-0.683	1.885	0.0058	0.2412	0.623	Down
112	7.181_303.2331334	CP 47,497-C6-Homolog		-0.682	1.892	0.0142	0.2898	0.623	Down
113	7.188_337.2589143	3-Sulfopyruvic acid	HMDB0004045	-0.678	2.015	0.0130	0.2847	0.625	Down
114	2.774_256.1188598	L-histidinol-phosphate	HMDB0304403	0.678	1.524	0.0457	0.3907	1.6	Up
115	7.182_304.2364782	4-Hydroxy Triamterene	HMDB0246440	-0.677	1.868	0.0141	0.2891	0.625	Down
116	6.030_267.1138580	Cyclonite	HMDB0253161	-0.677	1.941	0.0113	0.2756	0.626	Down

117	9.456_186.95 63442	Iodoacetic acid	HMDB0253518	-0.676	1.762	0.0373	0.3661	0.626	Down
118	8.172_898.62 25035	(S)-3-hydroxybutanoyl-CoA	HMDB0303997	-0.674	1.428	0.0199	0.3205	0.627	Down
119	7.486_499.37 95737	6"-Malonylcosmosiin	HMDB0037346	-0.663	1.761	0.0118	0.2777	0.631	Down
120	0.727_322.91 36518	Molybdate	HMDB0012260	-0.659	1.837	0.0233	0.3345	0.633	Down
121	0.616_129.01 93967	Mesaconic acid	HMDB0000749	-0.655	1.92	0.0061	0.2430	0.635	Down
122	6.490_374.29 86894	isoxicam	HMDB0253714	0.653	1.762	0.0385	0.3701	1.573	Up
123	4.205_238.10 68861	2-acetamido-3-(4-methoxyphenyl)propanoic acid		0.653	1.835	0.0208	0.3260	1.572	Up
124	7.448_398.37 42449	Luxabendazole	HMDB0254228	0.653	1.766	0.0188	0.3168	1.572	Up
125	5.235_292.15 40630	D-Glucuronic acid 1-phosphate	HMDB0003976	0.649	1.627	0.0218	0.3306	1.568	Up
126	0.605_227.95 49635	Pentaerythrityl tetrachloride	HMDB0256255	-0.647	1.997	0.0125	0.2805	0.639	Down
127	0.864_328.10 74022	N-Acetylneuraminic acid	HMDB0000230	0.638	1.638	0.0260	0.3432	1.556	Up

128	0.700_786.98 39832	Deacetylvinblastine	HMDB0250907	0.635	1.765	0.0392	0.3721	1.552	Up
129	5.166_145.14 16870	Disodium cyanodithioimidocarbamate	HMDB0303641	-0.633	1.853	0.0092	0.2628	0.645	Down
130	0.731_171.01 67443	Perflutren	HMDB0014696	0.633	1.926	0.0035	0.2298	1.551	Up
131	7.396_301.23 85802	1,3-Bis(4-nitrophenyl)urea	HMDB0244158	-0.632	1.807	0.0210	0.3280	0.645	Down
132	3.795_263.03 94561	Perfluorovaleric acid	HMDB0256331	0.631	1.551	0.0467	0.3916	1.548	Up
133	5.510_395.24 20290	Aldobiouronic acid	HMDB0302892	0.63	1.722	0.0280	0.3455	1.547	Up
134	3.054_201.11 32899	3-tert-Butyladipic acid		0.63	1.696	0.0416	0.3812	1.547	Up
135	0.644_145.09 81712	Methyl methanesulfonate	HMDB0254601	0.624	1.629	0.0129	0.2837	1.541	Up
136	0.606_111.00 87913	3-Furoic acid	HMDB0000444	-0.623	1.794	0.0076	0.2536	0.649	Down
137	7.184_321.21 01194	Pseudobaptigenin	HMDB0036616	-0.618	1.856	0.0120	0.2782	0.652	Down
138	0.594_148.11 62502	pyramid	HMDB0256941	0.615	1.886	0.0095	0.2628	1.531	Up
139	3.845_199.09 75715	3,5-Dinitrocatechol	HMDB0246069	-0.613	1.659	0.0175	0.3142	0.654	Down

140	4.665_165.09 10424	4-Phenylbutyric acid	HMDB0000543	0.609	1.832	0.0026	0.2288	1.525	Up
141	6.850_541.32 91182	Alinidine	HMDB0248138	-0.607	1.789	0.0055	0.2403	0.657	Down
142	3.993_108.02 17269	Dimethyl selenide	HMDB0033212	-0.606	1.796	0.0123	0.2792	0.657	Down
143	6.478_331.22 82284	17alpha-Hydroxypregnenolone	HMDB0000363	0.606	1.813	0.0238	0.3368	1.522	Up
144	0.713_171.03 60427	Oxalacetic acid	HMDB0000223	0.606	1.871	0.0267	0.3432	1.522	Up
145	0.727_520.91 00163	Afatinib	HMDB0248040	-0.601	1.8	0.0284	0.3460	0.659	Down
146	8.765_484.73 55914	6alpha-hydroxy-castasterone	HMDB0304235	-0.596	1.917	0.0064	0.2457	0.661	Down
147	9.587_639.10 41755	Perfluorodecane sulfonic acid	HMDB0246166	0.594	1.8	0.0416	0.3812	1.509	Up
148	0.717_676.72 06180	Rifamycins	HMDB0257226	-0.593	1.899	0.0001	0.1562	0.663	Down
149	6.782_1011.1 502069	CDP-DG(18:1(9Z)/20:4(8Z,11Z,14Z,17Z))	HMDB0007000	0.591	1.404	0.0320	0.3532	1.506	Up
150	0.595_147.11 29140	DL-Lysine	HMDB0142894	0.589	1.786	0.0255	0.3405	1.504	Up
151	6.668_253.12 67835	Clorgiline	HMDB0250365	0.589	1.615	0.0086	0.2563	1.504	Up

152	0.556_203.22 31813	1,9-Pyrazoloanthrone	HMDB0244499	0.589	1.771	0.0146	0.2956	1.504	Up
153	7.391_256.23 62526	Ibafloxacin	HMDB0253322	-0.588	1.637	0.0458	0.3907	0.665	Down
154	2.833_199.09 75885	3-Hydroxysebacic acid	HMDB0000350	0.588	1.708	0.0446	0.3888	1.503	Up
155	7.269_787.67 86055	Bapta-AM	HMDB0248864	-0.586	1.619	0.0321	0.3532	0.666	Down
156	1.537_134.10 61908	2,5-Dimethyl-1H-pyrrole	HMDB0032973	0.583	1.766	0.0249	0.3373	1.498	Up
157	0.640_502.94 32205	eribaxaban	HMDB0251919	0.583	1.809	0.0485	0.3961	1.498	Up
158	5.813_294.20 46397	Fenitrooxone	HMDB0252202	-0.58	1.772	0.0072	0.2531	0.669	Down
159	0.669_510.88 55799	Benoxaprofen glucuronide	HMDB0248957	0.576	1.727	0.0204	0.3236	1.49	Up
160	0.688_175.11 91077	DL-Arginine		0.574	1.727	0.0004	0.2062	1.489	Up
161	0.727_762.76 94333	N-Benzyladriamycin-14-valerate	HMDB0255092	-0.571	1.883	0.0029	0.2288	0.673	Down
162	0.687_176.12 24159	Pralidoxime	HMDB0014871	0.567	1.697	0.0003	0.2062	1.481	Up
163	2.390_248.03 28945	3-Chloro-2-hydroxypropanoic acid	HMDB0244784	0.561	1.795	0.0129	0.2837	1.475	Up

164	3.496_334.1394844	gamma-Glutamyltryptophan	HMDB0029160	0.561	1.792	0.0010	0.2280	1.475	Up
165	0.658_292.8065742	Pretilachlor	HMDB0256769	-0.558	1.774	0.0122	0.2792	0.679	Down
166	4.959_237.1089680	3,4-Bis(trifluoromethyl)-1H-pyrazole	HMDB0246017	0.558	1.695	0.0048	0.2403	1.472	Up
167	2.498_158.0822863	N-Isovalerylglycine	HMDB0000678	0.557	1.483	0.0289	0.3480	1.471	Up
168	0.661_768.9991260	PE(P-18:1(9Z)/18:3(9Z,12Z,15Z))	HMDB0011444	0.555	1.503	0.0208	0.3260	1.469	Up
169	5.054_228.1605568	2-((3,5-Dihydroxyphenyl)amino)acetic acid	HMDB0244908	0.55	1.393	0.0482	0.3959	1.464	Up
170	0.688_236.0209886	Bromobenzene-3,4-dihydrodiol	HMDB0060447	0.548	1.579	0.0426	0.3846	1.462	Up
171	0.553_195.2086726	2-[(Isopropylthio)methyl]furan	HMDB0036181	0.547	1.597	0.0301	0.3504	1.461	Up
172	7.099_452.2786983	LysoPE(0:0/16:0)	HMDB0011473	-0.546	1.482	0.0473	0.3941	0.685	Down
173	6.765_619.2911410	PI(18:1(9Z)/0:0)		-0.543	1.493	0.0323	0.3532	0.686	Down
174	5.861_277.1444845	Mono(2-ethylhexyl) phthalate (MEHP)	HMDB0013248	0.543	1.418	0.0466	0.3916	1.457	Up

175	1.112_129.0182266	Calcium chloride (CaCl2)	HMDB0303526	-0.543	1.622	0.0049	0.2403	0.686	Down
176	8.857_461.9881343	Clobetasone butyrate	HMDB0250333	0.541	1.678	0.0247	0.3373	1.455	Up
177	4.188_239.1099697	Fumaryl acetoacetate	HMDB0252519	0.54	1.691	0.0344	0.3564	1.454	Up
178	0.607_187.0725702	N-Acetyl-L-glutamine		0.54	1.775	0.0170	0.3131	1.454	Up
179	8.736_476.7489744	Diaryl ether	HMDB0251142	-0.54	1.839	0.0032	0.2288	0.688	Down
180	6.656_544.2961510	4-(cytidine 5'-diphospho)-2-C-methyl-D-erythritol	HMDB0304166	-0.539	1.606	0.0126	0.2808	0.688	Down
181	7.251_555.3510166	Estramustine phosphate	HMDB0251973	-0.538	1.412	0.0453	0.3907	0.689	Down
182	6.936_147.0856365	Acetic anhydride	HMDB0031646	-0.537	1.354	0.0271	0.3437	0.689	Down
183	7.008_565.4097305	p-MPPI	HMDB0256051	-0.536	1.68	0.0410	0.3796	0.689	Down
184	6.683_502.2934630	Fexofenadine	HMDB0005030	-0.534	1.619	0.0224	0.3330	0.69	Down
185	1.782_175.1190502	Oxonic Acid	HMDB0255998	0.532	1.302	0.0413	0.3807	1.446	Up
186	0.727_316.9481004	3,3',4,4'-Tetrachloroazoxybenzene	HMDB0246008	-0.532	1.79	0.0037	0.2298	0.692	Down

187	6.035_352.3185779	N-(4-Methylphenyl)diphenimide	HMDB0255014	0.527	1.47	0.0484	0.3959	1.441	Up
188	2.944_843.1929016	PC(22:6(4Z,7Z,10Z,13Z,16Z,19Z)/20:1(11Z))	HMDB0008735	0.527	1.808	0.0014	0.2288	1.441	Up
189	0.746_117.0751075	Dihydropyrimidine	HMDB0251326	0.526	1.736	0.0031	0.2288	1.44	Up
190	8.734_412.2715360	Altropane	HMDB0248262	-0.525	1.63	0.0086	0.2563	0.695	Down
191	6.003_287.2134724	4-Hydroxyvalerylcarnitine	HMDB0241653	-0.525	1.424	0.0179	0.3148	0.695	Down
192	5.105_269.1046866	3-deoxy-D-arabino-heptulosonate-7-phosphate	HMDB0304124	0.525	1.734	0.0095	0.2628	1.438	Up
193	1.894_89.0270473	formate	HMDB0304356	-0.523	1.594	0.0026	0.2288	0.696	Down
194	4.956_179.1066930	Selenomethionine	HMDB0003966	0.52	1.583	0.0104	0.2690	1.434	Up
195	8.165_719.5702902	6''-O-alpha-D-Galactopyranosylceritol	HMDB0036241	-0.519	1.625	0.0410	0.3798	0.698	Down
196	4.984_270.1156928	Deoxyinosine	HMDB0000071	0.516	1.542	0.0270	0.3437	1.43	Up

197	2.775_203.1608186	Methylhexahydrophthalic anhydride	HMDB0254649	0.514	1.49	0.0043	0.2376	1.428	Up
198	4.729_176.0786351	Salicylates	HMDB0302214	0.511	1.642	0.0017	0.2288	1.425	Up
199	8.650_792.0563146	SM(d17:1/5-iso PGF2VI)	HMDB0290370	-0.509	1.365	0.0343	0.3563	0.703	Down
200	0.909_136.0757377	N-Benzylformamide		0.509	1.73	0.0103	0.2683	1.423	Up
201	6.070_365.2774605	thioinosine monophosphate	HMDB0259014	-0.504	1.397	0.0496	0.3978	0.705	Down
202	0.685_162.9570546	3-Hexenedioic acid	HMDB0000393	0.504	1.627	0.0465	0.3916	1.418	Up
203	0.717_238.9316803	Trimetaphosphoric acid	HMDB0059921	-0.503	1.756	0.0020	0.2288	0.706	Down
204	0.721_479.0026030	Ponesimod	HMDB0256699	-0.501	1.477	0.0424	0.3836	0.707	Down
205	0.607_203.1037802	N6-Carboxymethyllysine	HMDB0240347	0.5	1.671	0.0132	0.2859	1.414	Up
206	8.906_182.0236796	6-Oxopiperidine-2-carboxylic acid	HMDB0061705	-0.499	1.723	0.0197	0.3188	0.708	Down
207	5.165_225.1244929	Pentobarbital	HMDB0014457	-0.498	1.51	0.0454	0.3907	0.708	Down
208	6.684_503.2967969	Ellagic acid glucoside	HMDB0301703	-0.494	1.537	0.0327	0.3532	0.71	Down

209	6.522_651.50 45803	Feruloyltartaric acid	HMDB0303700	0.491	1.534	0.0096	0.2642	1.406	Up
210	0.746_116.07 17108	5-Aminovaleric acid	HMDB0003355	0.49	1.697	0.0049	0.2403	1.404	Up
211	0.599_193.02 37128	Ethyl, 2,2,2-trifluoro-1-oxo-	HMDB0252063	-0.49	1.566	0.0167	0.3131	0.712	Down
212	7.904_352.25 54374	Narciclasine	HMDB0255458	-0.487	1.407	0.0363	0.3617	0.713	Down
213	2.715_130.05 90636	Cyanoacetic acid	HMDB0250607	0.486	1.623	0.0300	0.3503	1.401	Up
214	0.730_401.91 32718	Delmadinone acetate	HMDB0250972	-0.484	1.723	0.0083	0.2541	0.715	Down
215	1.514_133.10 53139	alpha-Fluoro-beta-ureidopropionic acid	HMDB0060435	0.478	1.658	0.0259	0.3432	1.393	Up
216	7.030_335.24 30431	gamma-Aminobutyric acid-betaxanthin	HMDB0304751	-0.478	1.552	0.0147	0.2959	0.718	Down
217	1.512_132.10 19703	L-Leucine	HMDB0000687	0.475	1.497	0.0300	0.3503	1.39	Up
218	6.771_1042.6 907137	TG(22:0/18:2(9Z,12Z)/22:2(13Z,16Z))	HMDB0046646	-0.47	1.351	0.0459	0.3907	0.722	Down
219	0.707_540.74 24940	N-Nervonoyl Glutamic acid	HMDB0242090	-0.47	1.685	0.0015	0.2288	0.722	Down

220	2.921_217.06 17516	1-(Difluoromethyl)-2,3,4-trifluorobenzene	HMDB0243802	0.465	1.557	0.0224	0.3330	1.38	Up
221	0.904_123.04 40669	Amitrole	HMDB0245804	0.463	1.621	0.0209	0.3260	1.378	Up
222	8.873_476.96 45797	Valechlorin	HMDB0041045	0.462	1.666	0.0031	0.2288	1.378	Up
223	0.704_418.99 89722	Zuclopenthixol	HMDB0015561	-0.461	1.685	0.0043	0.2364	0.726	Down
224	3.118_164.04 34863	Cyanuric acid	HMDB0041861	0.461	1.637	0.0378	0.3677	1.377	Up
225	3.115_163.04 01329	Phenylpyruvic acid	HMDB0000205	0.46	1.49	0.0139	0.2891	1.375	Up
226	1.513_86.096 4720	Piperidine	HMDB0034301	0.456	1.518	0.0293	0.3489	1.372	Up
227	8.835_142.94 94892	Caproic acid	HMDB0000535	-0.456	1.634	0.0231	0.3345	0.729	Down
228	2.502_160.08 64950	2-Amino-3,4-dihydroxypentanedioic acid	HMDB0030408	0.456	1.27	0.0443	0.3882	1.371	Up
229	0.941_161.08 19841	6-Methyluracil	HMDB0247093	0.454	1.439	0.0173	0.3131	1.37	Up
230	4.347_190.14 38595	Thieno[2,3-b]pyridin-4-ol	HMDB0258984	0.453	1.418	0.0150	0.2992	1.368	Up
231	6.841_337.27 35722	Dexbrompheniramine	HMDB0014549	0.452	1.56	0.0242	0.3368	1.368	Up

232	0.734_620.84 80605	DG(LTE4/0:0/8:0)	HMDB0297134	-0.452	1.697	0.0064	0.2457	0.731	Down
233	3.522_912.49 38086	MG(a-25:0/0:0/0:0)[rac]	HMDB0072857	0.451	1.505	0.0081	0.2536	1.367	Up
234	8.603_803.05 25366	PC(TXB2/14:0)	HMDB0285623	-0.449	1.372	0.0493	0.3975	0.733	Down
235	2.943_754.18 93251	DG(24:1n9/0:0/20:2n6)	HMDB0056270	0.449	1.393	0.0055	0.2403	1.365	Up
236	7.248_583.94 26739	Calcium palmitate	HMDB0303203	0.447	1.469	0.0496	0.3978	1.363	Up
237	8.916_149.99 74927	L-Serine	HMDB0000187	-0.443	1.629	0.0172	0.3131	0.736	Down
238	4.529_233.07 79954	3,5-Dichloro-L-tyrosine	HMDB0244684	0.442	1.545	0.0325	0.3532	1.359	Up
239	4.966_216.13 09827	4(1H)-Pteridinone, 2-amino-6-methyl-	HMDB0247091	0.441	1.578	0.0081	0.2536	1.357	Up
240	0.708_242.07 57554	4-Nitro-3-(trifluoromethyl)phenol	HMDB0246527	0.439	1.433	0.0263	0.3432	1.356	Up
241	0.858_166.05 33158	L-Methionine sulfoxide	HMDB0002005	-0.438	1.383	0.0211	0.3282	0.738	Down
242	6.184_309.32 63985	Taglutimida	HMDB0249202	-0.437	1.586	0.0224	0.3330	0.739	Down
243	6.955_597.92 48734	DG(16:1n7/0:0/16:1n7)	HMDB0056156	0.433	1.255	0.0477	0.3952	1.35	Up

244	0.756_241.0918662	Fructose-6-Phosphate (closed form)	HMDB0257695	0.433	1.299	0.0092	0.2628	1.35	Up
245	0.686_668.7866546	Janthitrem G	HMDB0030531	-0.431	1.276	0.0499	0.3978	0.742	Down
246	5.232_500.3099373	4-Chloro-5-sulfamoylanthranilic acid	HMDB0060761	-0.431	1.091	0.0492	0.3975	0.742	Down
247	4.416_188.1645065	4,6-Dihydroxy-2-quinolinecarboxylic acid	HMDB0033528	-0.43	1.532	0.0013	0.2288	0.742	Down
248	5.567_271.2740455	Glycyl-Tyrosine	HMDB0304778	0.429	1.498	0.0159	0.3039	1.347	Up
249	5.153_352.2454857	Cmp_257		-0.428	1.592	0.0120	0.2782	0.743	Down
250	7.013_761.5884671	Prunin 4",6"-di-O-gallate	HMDB0033652	-0.428	1.384	0.0381	0.3685	0.744	Down
251	4.420_952.4841887	TG(20:0/16:1(9Z)/20:0)	HMDB0045685	0.425	1.53	0.0005	0.2062	1.342	Up
252	0.716_692.6949333	Beclobrate	HMDB0248922	-0.425	1.375	0.0336	0.3546	0.745	Down
253	5.905_325.2009919	Imirestat	HMDB0253435	-0.424	1.368	0.0064	0.2457	0.745	Down
254	0.704_185.0979776	Benzoyl phosphate	HMDB0060440	-0.423	1.39	0.0483	0.3959	0.746	Down

255	0.678_608.8315858	lasalocid	HMDB0253985	-0.419	1.436	0.0025	0.2288	0.748	Down
256	7.014_760.5848769	PC(16:0/18:1(9Z))	HMDB0007972	-0.419	1.374	0.0341	0.3556	0.748	Down
257	7.025_661.4524890	Fodipir	HMDB0252426	-0.418	1.467	0.0227	0.3331	0.749	Down
258	2.299_190.0544236	2-(acetylamino)-4-(methylthio)butanoic acid		0.415	1.416	0.0180	0.3148	1.334	Up
259	2.775_204.0859154	Phosphodimethylethanolamine	HMDB0060244	0.414	1.466	0.0039	0.2298	1.332	Up
260	0.670_146.0653043	Nitroimidazopyran	HMDB0255652	0.414	1.227	0.0135	0.2886	1.332	Up
261	6.065_162.0996585	3-Nitrophenol	HMDB0245949	-0.409	1.478	0.0116	0.2777	0.753	Down
262	6.965_329.2474295	11(Z),14(Z),17(Z)-Eicosatrienoic acid	HMDB0060039	-0.409	1.412	0.0226	0.3330	0.753	Down
263	0.680_610.8282167	Dimyristoyl phosphatidic acid	HMDB0251413	-0.409	1.4	0.0168	0.3131	0.753	Down
264	5.173_144.1383767	Valpromide		-0.408	1.398	0.0195	0.3178	0.754	Down
265	2.786_165.0557607	L-(-)-3-Phenyllactic acid	HMDB0000563	0.407	1.607	0.0016	0.2288	1.326	Up
266	8.740_477.2506689	Z-Asp-CH2-DCB	HMDB0253346	-0.406	1.464	0.0181	0.3148	0.755	Down

267	0.607_247.0936014	Dicarbonyl methylglyoxal	HMDB0251183	0.406	1.504	0.0273	0.3437	1.325	Up
268	7.257_591.9320269	Muricatenol	HMDB0035900	-0.405	1.418	0.0350	0.3580	0.755	Down
269	3.054_172.0979893	N-Acetyl-D-alloisoleucine		0.405	1.456	0.0190	0.3168	1.324	Up
270	6.962_346.2739297	Diclobutrazol	HMDB0251205	-0.405	1.402	0.0107	0.2726	0.755	Down
271	6.674_544.3400070	LysoPC(0:0/20:4(5Z,8Z,11Z,14Z))	HMDB0061699	-0.405	1.419	0.0043	0.2376	0.755	Down
272	9.127_708.0390292	Cholesteryl (pyren-1-yl)hexanoate	HMDB0247687	0.402	1.361	0.0330	0.3540	1.321	Up
273	0.716_130.0622002	Creatine	HMDB0000064	0.398	1.295	0.0312	0.3528	1.318	Up
274	0.743_240.0805808	Platinum	HMDB0013671	0.391	1.348	0.0279	0.3446	1.311	Up
275	5.329_284.0959606	Ethacrynic acid	HMDB0015039	0.39	1.232	0.0383	0.3697	1.31	Up
276	0.677_761.0436304	Spirolide F	HMDB0030501	-0.389	1.312	0.0380	0.3679	0.763	Down
277	6.086_497.2500152	Ethene, 1,1-diphenyl-2,2-dichloro-	HMDB0252010	-0.389	1.284	0.0247	0.3373	0.763	Down
278	0.695_588.8467546	Cer(d16:1/18:1(9Z)-O(12,13))	HMDB0289866	-0.389	1.51	0.0100	0.2652	0.763	Down

279	6.587_239.2006356	N-alpha-acetyl lysine methyl ester	HMDB0304430	0.387	1.417	0.0468	0.3919	1.308	Up
280	2.514_176.1135982	serine thiol	HMDB0258249	-0.387	1.169	0.0152	0.3006	0.765	Down
281	7.087_244.1907422	Laurolactam		0.387	1.364	0.0136	0.2891	1.307	Up
282	8.792_818.6069142	PC(O-18:0/20:4(8Z,11Z,14Z,17Z))	HMDB0013420	-0.386	1.335	0.0187	0.3168	0.765	Down
283	7.486_657.5517008	ferroleghemoglobin	HMDB0304354	0.382	1.161	0.0133	0.2861	1.304	Up
284	6.445_460.2763736	1,2,3,4,6,7,8-Heptachlorodibenzo-P-dioxin	HMDB0253098	0.382	1.492	0.0019	0.2288	1.303	Up
285	0.740_116.0706903	D-(+)-Proline	HMDB0003411	0.382	1.077	0.0448	0.3892	1.303	Up
286	7.044_427.7079545	4,6-Hexacosanedione	HMDB0035554	0.377	1.298	0.0185	0.3168	1.299	Up
287	0.732_163.9779153	Taurine	HMDB0000251	0.377	1.33	0.0487	0.3970	1.298	Up
288	0.632_169.0949118	Pyridoxamine	HMDB0001431	0.376	1.297	0.0309	0.3522	1.298	Up
289	0.818_943.2251982	PE(PGF1alpha/24:0)	HMDB0284539	-0.376	1.345	0.0251	0.3395	0.771	Down
290	2.508_175.1779517	O-Phenolsulfonic acid	HMDB0304953	-0.373	1.444	0.0243	0.3368	0.772	Down

291	6.929_757.5546862	PE(16:0/20:4(5Z,8Z,11Z,14Z))	HMDB0008937	-0.372	1.342	0.0433	0.3856	0.773	Down
292	8.937_196.0030298	4-Hydroxyhippuric acid	HMDB0013678	-0.371	1.476	0.0220	0.3308	0.773	Down
293	6.840_788.9957356	PS(20:4(5Z,8Z,11Z,14Z)/14:0)	HMDB0012429	-0.366	1.346	0.0283	0.3460	0.776	Down
294	2.775_205.0893850	Gallic acid	HMDB0005807	0.366	1.235	0.0291	0.3489	1.289	Up
295	6.962_347.2774356	Delpazolid	HMDB0250974	-0.366	1.315	0.0157	0.3035	0.776	Down
296	8.736_413.7663498	Heptacosane	HMDB0302068	0.366	1.138	0.0176	0.3142	1.289	Up
297	0.697_157.0803095	(S)-Ureidoglycolic acid	HMDB0001005	0.364	1.2	0.0064	0.2457	1.287	Up
298	2.943_842.1908437	Testosterone phenylpropionate	HMDB0258861	0.362	1.463	0.0056	0.2403	1.286	Up
299	6.947_325.7549579	Clofop	HMDB0250346	0.362	1.129	0.0399	0.3750	1.285	Up
300	7.024_661.9543220	Furazabol	HMDB0252534	-0.36	1.325	0.0477	0.3952	0.779	Down
301	0.749_90.0550534	beta-Alanine	HMDB0000056	0.359	1.113	0.0423	0.3836	1.283	Up
302	0.727_788.7726261	Loteprednol	HMDB0015011	0.359	1.149	0.0142	0.2891	1.283	Up

303	5.488_225.1485772	3-hydroxy-2-octylpentanedioic acid		0.358	1.324	0.0389	0.3720	1.281	Up
304	8.440_854.5677680	PC(18:1(9Z)/22:6(4Z,7Z,10Z,13Z,16Z,19Z))	HMDB0008123	-0.356	1.456	0.0024	0.2288	0.781	Down
305	6.275_207.0929895	Chelidonic acid	HMDB0249899	0.353	1.303	0.0219	0.3306	1.278	Up
306	4.845_222.1135827	Cerulenin	HMDB0015168	0.353	1.331	0.0314	0.3532	1.277	Up
307	0.721_618.7615183	M8-Nelfinavir	HMDB0255505	-0.345	1.416	0.0094	0.2628	0.787	Down
308	0.831_315.0603555	Perfluorohexanoic acid	HMDB0256324	-0.345	1.409	0.0004	0.2062	0.787	Down
309	5.946_715.0405614	SM C16:1	HMDB0029216	-0.344	1.316	0.0124	0.2792	0.788	Down
310	2.116_131.0713423	5-Hydroxyhexanoic acid	HMDB0000525	0.343	1.265	0.0159	0.3035	1.268	Up
311	0.688_269.0876800	Hydroxyaniline mustard	HMDB0253255	0.338	1.325	0.0118	0.2777	1.264	Up
312	1.855_215.0328484	D-Psicose	HMDB0250793	-0.337	1.107	0.0462	0.3910	0.792	Down
313	8.931_176.9594943	Terbium	HMDB0303932	-0.331	1.437	0.0087	0.2579	0.795	Down
314	2.943_144.0808088	2-Naphthylamine	HMDB0041802	0.328	1.261	0.0037	0.2298	1.255	Up

315	6.592_497.33 24734	Sodium glucoheptonate	HMDB0303297	-0.327	1.297	0.0259	0.3432	0.797	Down
316	6.666_567.32 51797	Etoprine	HMDB0252116	-0.325	1.329	0.0020	0.2288	0.798	Down
317	2.800_296.97 98777	Glucose 1-phosphate	HMDB0001586	-0.325	1.279	0.0388	0.3720	0.799	Down
318	2.943_143.07 25145	(S)-2-Hydrazinopropionic acid	HMDB0248305	0.323	1.197	0.0072	0.2527	1.251	Up
319	2.943_132.08 07560	Skatole	HMDB0000466	0.321	1.22	0.0052	0.2403	1.249	Up
320	4.976_232.15 44632	Pyridoxine 5'-phosphate	HMDB0001319	0.32	1.187	0.0375	0.3669	1.248	Up
321	4.758_222.14 61722	4-Fluoro-L-phenylalanine	HMDB0246423	-0.32	1.258	0.0179	0.3148	0.801	Down
322	5.084_224.17 27104	4-(Phosphonomethyl) piperidine-2-carboxylic acid	HMDB0246313	0.319	1.278	0.0434	0.3861	1.247	Up
323	0.643_314.06 89196	Alcoifosfamide	HMDB0060693	0.317	1.312	0.0355	0.3605	1.246	Up
324	5.183_351.23 34080	2-O-(beta-L-Arabinopyranosyl)-myoinositol	HMDB0302392	-0.315	1.353	0.0312	0.3528	0.804	Down
325	5.795_334.23 55056	2-Methacryloyloxyeth	HMDB0247194	-0.313	1.332	0.0438	0.3872	0.805	Down

		yl phosphorylcholine							
326	0.886_111.05 53227	Glyoxime	HMDB0252890	0.311	1.224	0.0469	0.3926	1.241	Up
327	7.028_303.21 70151	9-(2,6-Dioxo-3H-purin-9-yl)-3H-purine-2,6-dione	HMDB0257773	-0.31	1.2	0.0361	0.3617	0.806	Down
328	0.759_338.06 51356	4,4'-Dichlorodiphenyldichloroethane	HMDB0246601	-0.309	1.367	0.0098	0.2651	0.807	Down
329	8.677_156.07 69299	L-Histidine	HMDB0000177	-0.309	1.345	0.0082	0.2541	0.807	Down
330	2.943_170.06 00590	3-Amino-2-naphthoic acid		0.307	1.198	0.0070	0.2507	1.237	Up
331	5.233_230.14 68078	5-Phenyl-1,3-oxazinane-2,4-dione	HMDB0060400	0.301	1.154	0.0467	0.3916	1.232	Up
332	7.028_320.19 78276	2-Chlorodiazepam	HMDB0251203	-0.301	1.148	0.0305	0.3514	0.812	Down
333	7.265_789.61 04328	Diflufenican	HMDB0251262	-0.301	1.211	0.0218	0.3306	0.812	Down
334	2.559_231.13 40058	Scopoletin	HMDB0034344	0.3	1.081	0.0119	0.2782	1.231	Up
335	0.677_240.83 89996	Thallium	HMDB0013724	-0.299	1.39	0.0017	0.2288	0.813	Down
336	4.440_306.26 32535	Sulfatroxazole	HMDB0258583	0.299	1.186	0.0193	0.3178	1.231	Up

337	6.075_254.24 78293	Fenfluramine	HMDB0252200	-0.299	1.225	0.0420	0.3833	0.813	Down
338	6.598_495.32 78362	Testosterone undecanoate	HMDB0002705	-0.299	1.256	0.0265	0.3432	0.813	Down
339	3.867_170.13 90220	beta-alanine betaine	HMDB0304538	0.293	1.214	0.0196	0.3187	1.225	Up
340	2.943_171.06 34366	Calcium phosphinate	HMDB0303421	0.293	1.12	0.0309	0.3522	1.225	Up
341	6.685_549.29 82230	Glucose-uridine-C1,5'-diphosphate	HMDB0259387	0.292	1.077	0.0423	0.3836	1.224	Up
342	8.860_359.00 70647	3,6-dichlorocatechol	HMDB0304104	0.29	1.228	0.0403	0.3766	1.223	Up
343	2.389_164.07 17605	L-Phenylalanine	HMDB0000159	0.288	1.247	0.0445	0.3888	1.221	Up
344	7.286_809.08 57318	PE-NMe(22:6(4Z,7Z,10Z,13Z,16Z,19Z)/16:1(9Z))	HMDB0113677	-0.285	1.154	0.0430	0.3851	0.821	Down
345	6.074_240.23 23120	5-(Tetrahydro-2H-pyran-4-yl)-1H-indole	HMDB0246737	0.283	1.099	0.0422	0.3836	1.217	Up
346	0.650_618.85 99840	PE(14:0/14:0)	HMDB0008821	0.281	1.223	0.0181	0.3149	1.215	Up
347	3.102_785.15 64889	nonaprenyl-4-hydroxybenzoate	HMDB0304438	0.275	1.077	0.0242	0.3368	1.21	Up

348	0.616_88.0400040	DL-Alanine	HMDB0062251	0.273	1.024	0.0161	0.3049	1.208	Up
349	3.503_458.2573905	CDP-N-methylethanolamine	HMDB0304287	0.266	1.134	0.0415	0.3812	1.203	Up

Significantly altered plasma metabolites in PTZ-kindled mice compared with vehicle-treated controls (PTZ vs CON, n = 6 per group). A total of 349 metabolites were identified as significantly altered based on the following criteria: VIP ≥ 1 derived from the OPLS-DA model, fold change ≥ 1.2 or ≤ 0.83, and $P < 0.05$ by two-tailed Student's *t*-test. Log2FC represents the log2-transformed fold change of the PTZ group relative to the CON group; positive values indicate upregulation and negative values indicate downregulation. Q values were calculated using the Benjamini-Hochberg method and are provided for reference. "Regulation" indicates the direction of change (Up or Down). HMDB identifiers are provided where available. Metabolites are sorted by descending absolute Log2FC value.

Abbreviations: FC, fold change; VIP, variable importance in projection; OPLS-DA, orthogonal partial least squares discriminant analysis; HMDB, Human Metabolome Database; PTZ, pentylenetetrazole; CON, control.

Supplementary Table S4. Annotation information and evidence-based classification of the six metabolic features retained by LASSO.

Putative annotation	HMDB accession	Log2FC(PTZ-vs-CON)	*P* value	LASSO coefficient	Annotation evidence/confidence	Possible origin or association	Supporting references
Indolelactic acid	HMDB0000671	0.853	<0.0001	-0.736	Vendor-provided putative tandem mass spectrometry (MS/MS) annotation; no authentic-standard confirmation	Tryptophan-derived metabolite with possible host and microbial origins	(Wang et al., 2024, Dodd et al., 2017)
DG(20:3n6/0:0/20:3n6)	HMDB0056321	3.815	<0.0001	-1.7579	Vendor-provided putative lipid annotation; exact isomeric structure unconfirmed	Glycerolipid-associated feature	(Conroy et al., 2024, Köfeler et al., 2021)
Rifamycins	HMDB0257226	-0.593	0.0001	0.0637	Vendor-provided class-level annotation; exact compound unconfirmed	Drug- or microbial-natural-product-associated; actual origin uncertain	(Qi et al., 2018)
3-Oxobrimonidine	HMDB0061102	0.974	0.0010	-0.0347	Vendor-provided putative annotation; no authentic-standard confirmation	Brimonidine-associated drug-metabolite annotation; no documented exposure	(Acheampong et al., 1996)
Pyrogallol-2-O-sulphate	HMDB0060018	-2.87	0.0016	0.0571	Vendor-provided putative annotation; no authentic-standard confirmation	Diet-, microbial-, and host-conjugation-associated; actual source uncertain	(van der Hooft et al., 2012)
Mometasone	HMDB0014902	-1.073	0.0009	0.2685	Vendor-provided putative annotation; no authentic-standard	Drug-associated corticosteroid	(Kim et al., 2025)

**Putative annotation**	**HMDB accession**	**Log2FC(PTZ-vs-CON)**	***P* value**	**LASSO coefficient**	**Annotation evidence/confidence**	**Possible origin or association**	**Supporting references**
					confirmation	annotation; no documented exposure	

All six metabolic features retained by LASSO (least absolute shrinkage and selection operator) are reported without post hoc exclusion. The possible-origin categories summarize biological, microbial, dietary, natural-product, or drug-related associations reported in databases and the literature; they do not establish the actual sources of these circulating features in the mice examined in this study. The HMDB accession numbers correspond to the vendor-provided putative annotations and do not constitute definitive compound identification(Wishart et al., 2022). HMDB records were accessed on July 17, 2026. Information regarding confirmation with authentic standards was not provided in the vendor report.

References for Supplementary Table S4:

[1]Acheampong AA, Chien DS, Lam S, Vekich S, Breau A, Usansky J, et al. 1996. Characterization of brimonidine metabolism with rat, rabbit, dog, monkey and human liver fractions and rabbit liver aldehyde oxidase. *Xenobiotica; the Fate of Foreign Compounds In Biological Systems,* 26**,** 1035-1055.

[2]Conroy MJ, Andrews RM, Andrews S, Cockayne L, Dennis EA, Fahy E, et al. 2024. LIPID MAPS: update to databases and tools for the lipidomics community. *Nucleic Acids Research,* 52**,** D1677-D1682. https://doi.org/10.1093/nar/gkad896

[3]Dodd D, Spitzer MH, Van Treuren W, Merrill BD, Hryckowian AJ, Higginbottom SK, et al. 2017. A gut bacterial pathway metabolizes aromatic amino acids into nine circulating metabolites. *Nature,* 551**,** 648-652. https://doi.org/10.1038/nature24661

[4]Kim S, Chen J, Cheng T, Gindulyte A, He J, He S, et al. 2025. PubChem 2025 update. *Nucleic Acids Research,* 53**,** D1516-D1525. https://doi.org/10.1093/nar/gkae1059

[5]Köfeler HC, Ahrends R, Baker ES, Ekroos K, Han X, Hoffmann N, et al. 2021. Recommendations for good practice in MS-based lipidomics. *Journal of Lipid Research,* 62**,** 100138. https://doi.org/10.1016/j.jlr.2021.100138

[6]Qi F, Lei C, Li F, Zhang X, Wang J, Zhang W, et al. 2018. Deciphering the late steps of rifamycin biosynthesis. *Nature Communications,* 9**,** 2342. https://doi.org/10.1038/s41467-018-04772-x

[7]van der Hooft JJJ, de Vos RCH, Mihaleva V, Bino RJ, Ridder L, de Roo N, et al. 2012. Structural elucidation and quantification of phenolic conjugates present in human urine after tea intake. *Analytical Chemistry,* 84**,** 7263-7271. https://doi.org/10.1021/ac3017339

[8]Wang G, Fan Y, Zhang G, Cai S, Ma Y, Yang L, et al. 2024. Microbiota-derived indoles alleviate intestinal inflammation and modulate microbiome by microbial cross-feeding. *Microbiome,* 12**,** 59. https://doi.org/10.1186/s40168-024-01750-y

[9]Wishart DS, Guo A, Oler E, Wang F, Anjum A, Peters H, et al. 2022. HMDB 5.0: the Human Metabolome Database for 2022. *Nucleic Acids Research,* 50**,** D622-D631. https://doi.org/10.1093/nar/gkab1062

Supplementary Table S5. Characteristics of the GSE256068 samples included in the human transcriptomic analysis.

GEO accession	Sample ID	Group	Disease subtype	Tissue in GEO	Sex	Age (years)	Seizure frequency
GSM8084989	TLE-HS_2	TLE-HS	TLE-HS	Temporal	Male	41	5
GSM8085002	TLE-HS_4	TLE-HS	TLE-HS	Temporal	Male	38	6
GSM8085006	TLE-HS_5	TLE-HS	TLE-HS	Temporal	Male	41	30
GSM8085008	TLE-HS_6	TLE-HS	TLE-HS	Temporal	Female	60	3
GSM8085014	TLE-HS_8	TLE-HS	TLE-HS	Temporal	Female	58	6
GSM8085020	TLE-HS_10	TLE-HS	TLE-HS	Temporal	Male	29	15
GSM8085021	TLE-HS_11	TLE-HS	TLE-HS	Temporal	Male	25	5
GSM8085022	TLE-HS_12	TLE-HS	TLE-HS	Temporal	Female	22	32
GSM8085023	TLE-HS_13	TLE-HS	TLE-HS	Temporal	Male	38	4
GSM8085027	TLE-HS_14	TLE-HS	TLE-HS	Temporal	Male	43	5
GSM8085033	TLE-HS_15	TLE-HS	TLE-HS	Temporal	Female	19	5
GSM8085038	TLE-HS_16	TLE-HS	TLE-HS	Temporal	Female	46	4
GSM8085039	TLE-HS_17	TLE-HS	TLE-HS	Temporal	Male	34	60
GSM8085041	TLE-HS_18	TLE-HS	TLE-HS	Temporal	Male	14	9
GSM8085042	TLE-HS_19	TLE-HS	TLE-HS	Temporal	Male	56	25
GSM8085043	TLE-HS_20	TLE-HS	TLE-HS	Temporal	Female	42	1
GSM8085046	TLE-HS_21	TLE-HS	TLE-HS	Temporal	Male	62	6
GSM8085049	TLE-HS_22	TLE-HS	TLE-HS	Temporal	Male	39	4
GSM8085050	TLE-HS_23	TLE-HS	TLE-HS	Temporal	Female	21	90
GSM8085051	TLE-HS_24	TLE-HS	TLE-HS	Temporal	Female	16	17

GEO accession	Sample ID	Group	Disease subtype	Tissue in GEO	Sex	Age (years)	Seizure frequency
GSM8085052	TLE-HS_25	TLE-HS	TLE-HS	Temporal	Male	49	5
GSM8085053	TLE-HS_26	TLE-HS	TLE-HS	Temporal	Male	9	7
GSM8085054	TLE-HS_27	TLE-HS	TLE-HS	Temporal	Female	17	2
GSM8085055	TLE-HS_28	TLE-HS	TLE-HS	Temporal	Male	24	5
GSM8085056	TLE-HS_29	TLE-HS	TLE-HS	Temporal	Female	19	17
GSM8085059	TLE-HS_30	TLE-HS	TLE-HS	Temporal	Male	14	17
GSM8085061	TLE-HS_31	TLE-HS	TLE-HS	Temporal	Male	29	36
GSM8085062	TLE-HS_32	TLE-HS	TLE-HS	Temporal	Female	35	30
GSM8085065	TLE-HS_34	TLE-HS	TLE-HS	Temporal	Female	47	3
GSM8085066	TLE-HS_35	TLE-HS	TLE-HS	Temporal	Female	39	5
GSM8085069	TLE-HS_36	TLE-HS	TLE-HS	Temporal	Female	22	243
GSM8085070	TLE-HS_37	TLE-HS	TLE-HS	Temporal	Male	58	4
GSM8085071	TLE-HS_38	TLE-HS	TLE-HS	Temporal	Male	55	3
GSM8085072	TLE-HS_39	TLE-HS	TLE-HS	Temporal	Male	43	8
GSM8085073	TLE-HS_40	TLE-HS	TLE-HS	Temporal	Male	58	30
GSM8085076	TLE-HS_41	TLE-HS	TLE-HS	Temporal	Male	60	20
GSM8085077	TLE-HS_42	TLE-HS	TLE-HS	Temporal	Male	19	5
GSM8085078	TLE-HS_43	TLE-HS	TLE-HS	Temporal	Male	41	4
GSM8085079	TLE-HS_44	TLE-HS	TLE-HS	Temporal	Male	43	5
GSM8085083	TLE-HS_45	TLE-HS	TLE-HS	Temporal	Female	36	6
GSM8085086	TLE-HS_46	TLE-HS	TLE-HS	Temporal	Female	27	5

GEO accession	Sample ID	Group	Disease subtype	Tissue in GEO	Sex	Age (years)	Seizure frequency
GSM8085087	TLE-HS_47	TLE-HS	TLE-HS	Temporal	Female	54	20
GSM8085090	TLE-HS_48	TLE-HS	TLE-HS	Temporal	Male	53	30
GSM8085091	TLE-HS_49	TLE-HS	TLE-HS	Temporal	Male	2	17
GSM8085094	TLE-HS_50	TLE-HS	TLE-HS	Temporal	Male	29	12
GSM8085095	TLE-HS_51	TLE-HS	TLE-HS	Temporal	Male	53	60
GSM8085096	TLE-HS_52	TLE-HS	TLE-HS	Temporal	Male	41	20
GSM8085097	TLE-HS_53	TLE-HS	TLE-HS	Temporal	Female	22	9
GSM8085098	TLE-HS_54	TLE-HS	TLE-HS	Temporal	Male	31	20
GSM8085101	TLE-HS_55	TLE-HS	TLE-HS	Temporal	Female	31	10
GSM8085102	TLE-HS_56	TLE-HS	TLE-HS	Temporal	Male	44	13
GSM8085104	TLE-HS_57	TLE-HS	TLE-HS	Temporal	Male	39	3
GSM8085105	TLE-HS_58	TLE-HS	TLE-HS	Temporal	Male	27	3
GSM8085106	TLE-HS_59	TLE-HS	TLE-HS	Temporal	Male	51	200
GSM8085109	TLE-HS_60	TLE-HS	TLE-HS	Temporal	Female	6	20
GSM8085110	TLE-HS_61	TLE-HS	TLE-HS	Temporal	Male	18	43
GSM8085111	TLE-HS_62	TLE-HS	TLE-HS	Temporal	Female	38	40
GSM8085115	TLE-HS_63	TLE-HS	TLE-HS	Temporal	Male	8	91
GSM8085125	TLE-HS_64	TLE-HS	TLE-HS	Temporal	Female	38	7
GSM8084991	ControlHippocampus_1	Control	Control hippocampus	Hippocampus	Male	69	NA
GSM8085009	ControlHippocampus_2	Control	Control hippocampus	Hippocampus	Male	49	NA
GSM8085018	ControlHippocampus_3	Control	Control hippocampus	Hippocampus	Female	82	NA

GEO accession	Sample ID	Group	Disease subtype	Tissue in GEO	Sex	Age (years)	Seizure frequency
GSM8085034	ControlHippocampus_4	Control	Control hippocampus	Hippocampus	Female	67	NA
GSM8085036	ControlHippocampus_5	Control	Control hippocampus	Hippocampus	Female	63	NA
GSM8085048	ControlHippocampus_6	Control	Control hippocampus	Hippocampus	Male	75	NA
GSM8085081	ControlHippocampus_8	Control	Control hippocampus	Hippocampus	Male	47	NA
GSM8085126	ControlHippocampus_10	Control	Control hippocampus	Hippocampus	Male	62	NA
GSM8085127	ControlHippocampus_11	Control	Control hippocampus	Hippocampus	Male	30	NA
GSM8085128	ControlHippocampus_12	Control	Control hippocampus	Hippocampus	Male	72	NA
GSM8085129	ControlHippocampus_13	Control	Control hippocampus	Hippocampus	Female	44	NA

Characteristics of the GSE256068 samples included in the complementary human transcriptomic analysis. The analysis included 59 samples from patients with temporal lobe epilepsy with hippocampal sclerosis (TLE-HS) and 11 control hippocampal samples. Age, sex, tissue label, disease state, and seizure-frequency information were obtained from the sample-level metadata deposited in the Gene Expression Omnibus. NA, not available or not applicable.

Supplementary Table S6. Differential-expression results for representative metabolism-related genes in human TLE-HS hippocampus (GSE256068, 59 TLE-HS vs 11 controls).

Gene	baseMean	Log2FC	*P*value	*P*adj	*P*
PHGDH	8330	0.6	0.000024	0.000171	***
PSAT1	6832.6	0.329	0.00342	0.0124	*
GAD1	3315	0.853	0.00000533	0.0000449	****
FASN	10292.7	0.513	0.00000102	0.0000102	****
TDO2	40.4	0.664	0.0175	0.0484	*

Differential-expression analysis was performed using DESeq2. baseMean, mean normalized count across all samples; Log2FC, $\log_2$ fold change (TLE-HS vs Control); *P*adj, adjusted *P* value (Benjamini–Hochberg); *P*, significance level (**** *P*adj < 0.0001, *** *P*adj < 0.001, * *P*adj < 0.05). All five genes showed significant upregulation in TLE-HS hippocampus. Positive log2FC values indicate higher expression in TLE-HS.

Supplementary Table S7. KEGG pathway enrichment results for differentially expressed genes between TLE-HS and control hippocampal samples (GSE256068, 59 TLE-HS vs 11 controls).

ID	Description	GeneRatio	BgRatio	*P*value	*P*adj	Qvalue	Count
hsa04080	Neuroactive ligand-receptor interaction	122/1328	371/9416	0	0	0	122
hsa04082	Neuroactive ligand signaling	70/1328	199/9416	0	0	0	70
hsa04060	Cytokine-cytokine receptor interaction	88/1328	298/9416	0	0	0	88
hsa04518	Integrin signaling	52/1328	154/9416	0	0	0	52
hsa04151	PI3K-Akt signaling pathway	94/1328	362/9416	0	0	0	94
hsa04820	Cytoskeleton in muscle cells	67/1328	233/9416	0	0	0	67
hsa04512	ECM-receptor interaction	35/1328	89/9416	0	0	0	35
hsa04610	Complement and coagulation cascades	34/1328	89/9416	0	0	0	34
hsa05150	Staphylococcus aureus infection	36/1328	99/9416	0	0	0	36
hsa04640	Hematopoietic cell lineage	36/1328	100/9416	0	0	0	36
hsa04020	Calcium signaling pathway	68/1328	254/9416	0	0	0	68

hsa04061	Viral protein interaction with cytokine and cytokine receptor	33/1328	100/9416	0	0	0	33
hsa04940	Type I diabetes mellitus	19/1328	44/9416	0	0.0001	0.0001	19
hsa04380	Osteoclast differentiation	41/1328	142/9416	0	0.0001	0.0001	41
hsa04668	TNF signaling pathway	36/1328	119/9416	0	0.0001	0.0001	36
hsa04510	Focal adhesion	52/1328	203/9416	0	0.0002	0.0001	52
hsa04933	AGE-RAGE signaling pathway in diabetic complications	31/1328	101/9416	0	0.0003	0.0002	31
hsa05033	Nicotine addiction	17/1328	41/9416	0	0.0003	0.0002	17
hsa05323	Rheumatoid arthritis	29/1328	95/9416	0	0.0005	0.0004	29
hsa04015	Rap1 signaling pathway	52/1328	212/9416	0	0.0005	0.0004	52
hsa05414	Dilated cardiomyopathy	31/1328	105/9416	0	0.0005	0.0004	31
hsa04081	Hormone signaling	53/1328	219/9416	0	0.0006	0.0005	53
hsa04727	GABAergic synapse	27/1328	89/9416	0.0001	0.0009	0.0007	27
hsa05410	Hypertrophic cardiomyopathy	29/1328	99/9416	0.0001	0.0009	0.0007	29
hsa05332	Graft-versus-host disease	17/1328	45/9416	0.0001	0.0009	0.0007	17
hsa04672	Intestinal immune network for IgA production	18/1328	50/9416	0.0001	0.0011	0.0009	18
hsa05144	Malaria	18/1328	50/9416	0.0001	0.0011	0.0009	18
hsa04611	Platelet activation	34/1328	126/9416	0.0001	0.0012	0.0009	34
hsa05133	Pertussis	24/1328	78/9416	0.0001	0.0014	0.0011	24
hsa05146	Amoebiasis	29/1328	103/9416	0.0001	0.0016	0.0013	29
hsa05321	Inflammatory bowel disease	21/1328	66/9416	0.0002	0.002	0.0016	21
hsa04926	Relaxin signaling pathway	34/1328	130/9416	0.0002	0.0021	0.0016	34
hsa05418	Fluid shear stress and atherosclerosis	36/1328	142/9416	0.0002	0.0026	0.002	36
hsa04974	Protein digestion and absorption	28/1328	103/9416	0.0003	0.0035	0.0027	28
hsa04620	Toll-like receptor signaling pathway	29/1328	109/9416	0.0004	0.004	0.0031	29
hsa05171	Coronavirus disease	53/1328	239/9416	0.0004	0.004	0.0031	53
hsa04064	NF-kappa B signaling pathway	28/1328	105/9416	0.0005	0.0046	0.0035	28

hsa05330	Allograft rejection	14/1328	39/9416	0.0005	0.005	0.0038	14
hsa00500	Starch and sucrose metabolism	13/1328	36/9416	0.0008	0.0071	0.0055	13
hsa05310	Asthma	12/1328	32/9416	0.0009	0.0075	0.0057	12
hsa05168	Herpes simplex virus 1 infection	41/1328	180/9416	0.001	0.0088	0.0067	41
hsa04514	Cell adhesion molecule (CAM) interaction	37/1328	160/9416	0.0013	0.0109	0.0083	37
hsa05165	Human papillomavirus infection	67/1328	333/9416	0.0014	0.0109	0.0083	67
hsa04713	Circadian entrainment	25/1328	97/9416	0.0016	0.0128	0.0098	25
hsa05140	Leishmaniasis	21/1328	78/9416	0.0021	0.0161	0.0123	21
hsa04625	C-type lectin receptor signaling pathway	26/1328	105/9416	0.0025	0.0182	0.0139	26
hsa04923	Regulation of lipolysis in adipocytes	17/1328	59/9416	0.0025	0.0182	0.0139	17
hsa04010	MAPK signaling pathway	60/1328	300/9416	0.0027	0.0196	0.0149	60
hsa05416	Viral myocarditis	19/1328	70/9416	0.003	0.0213	0.0163	19
hsa04662	B cell receptor signaling pathway	23/1328	91/9416	0.0032	0.0218	0.0166	23
hsa05032	Morphine addiction	23/1328	91/9416	0.0032	0.0218	0.0166	23
hsa05412	Arrhythmogenic right ventricular cardiomyopathy	22/1328	86/9416	0.0033	0.0221	0.0169	22
hsa05143	African trypanosomiasis	12/1328	37/9416	0.0036	0.0236	0.018	12
hsa00603	Glycosphingolipid biosynthesis - globo and isoglobo series	7/1328	16/9416	0.0039	0.025	0.0191	7
hsa04658	Th1 and Th2 cell differentiation	23/1328	93/9416	0.0043	0.027	0.0206	23
hsa05224	Breast cancer	33/1328	148/9416	0.0044	0.027	0.0206	33
hsa04145	Phagocytosis	52/1328	259/9416	0.0046	0.0281	0.0215	52
hsa04750	Inflammatory mediator regulation of TRP channels	24/1328	99/9416	0.0047	0.0281	0.0215	24
hsa05202	Transcriptional misregulation in cancer	42/1328	201/9416	0.005	0.0295	0.0225	42
hsa05164	Influenza A	37/1328	173/9416	0.0055	0.0318	0.0243	37
hsa04024	cAMP signaling pathway	46/1328	226/9416	0.0058	0.0325	0.0248	46
hsa04014	Ras signaling pathway	48/1328	238/9416	0.0058	0.0325	0.0248	48
hsa04270	Vascular smooth muscle contraction	30/1328	134/9416	0.006	0.033	0.0252	30

hsa05166	Human T-cell leukemia virus 1 infection	45/1328	224/9416	0.008	0.0431	0.0329	45
hsa04723	Retrograde endocannabinoid signaling	32/1328	149/9416	0.0088	0.047	0.0359	32

Over-representation analysis was performed using the enrich Kyoto Encyclopedia of Genes and Genomes (KEGG) function in the clusterProfiler package in R. GeneRatio, number of differentially expressed genes in the pathway / total number of differentially expressed genes; BgRatio, number of genes in the pathway / total number of background genes; *P*adj, adjusted *P* value (Benjamini–Hochberg); Count, number of differentially expressed genes mapped to the pathway. Pathways are sorted by ascending p.adjust value.

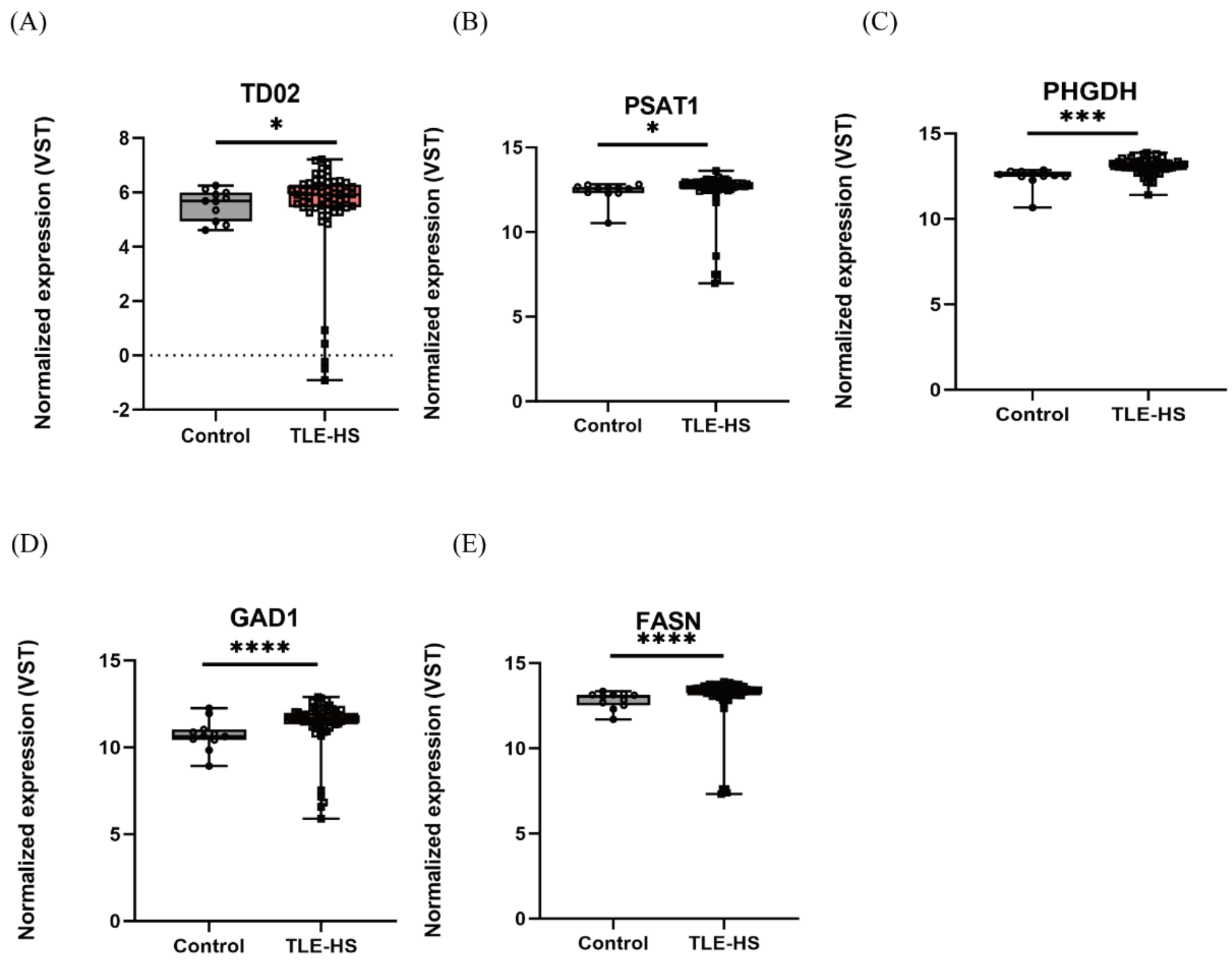

Supplementary Figure S1. Boxplots of variance-stabilized expression values for representative metabolism-related genes in human TLE-HS hippocampus (GSE256068, 59 TLE-HS vs 11 controls). Five genes mapping to the key metabolic pathways identified in the mouse plasma metabolomic analysis were examined: PHGDH and PSAT1 (serine biosynthesis), GAD1 ((glutamate–gamma-aminobutyric acid (GABA) metabolism)), FASN (fatty acid synthesis), and TDO2 (tryptophan–kynurenine metabolism). Expression values were extracted from the DESeq2-processed dataset using variance-stabilizing transformation (VST). Boxplots were generated in GraphPad Prism (version 10.1.2). All five genes showed significant upregulation in TLE-HS hippocampus ($P < 0.05$, DESeq2). No additional statistical tests were applied to the plotted expression values. TLE-HS, temporal lobe epilepsy with hippocampal sclerosis; VST, variance-stabilizing transformation.

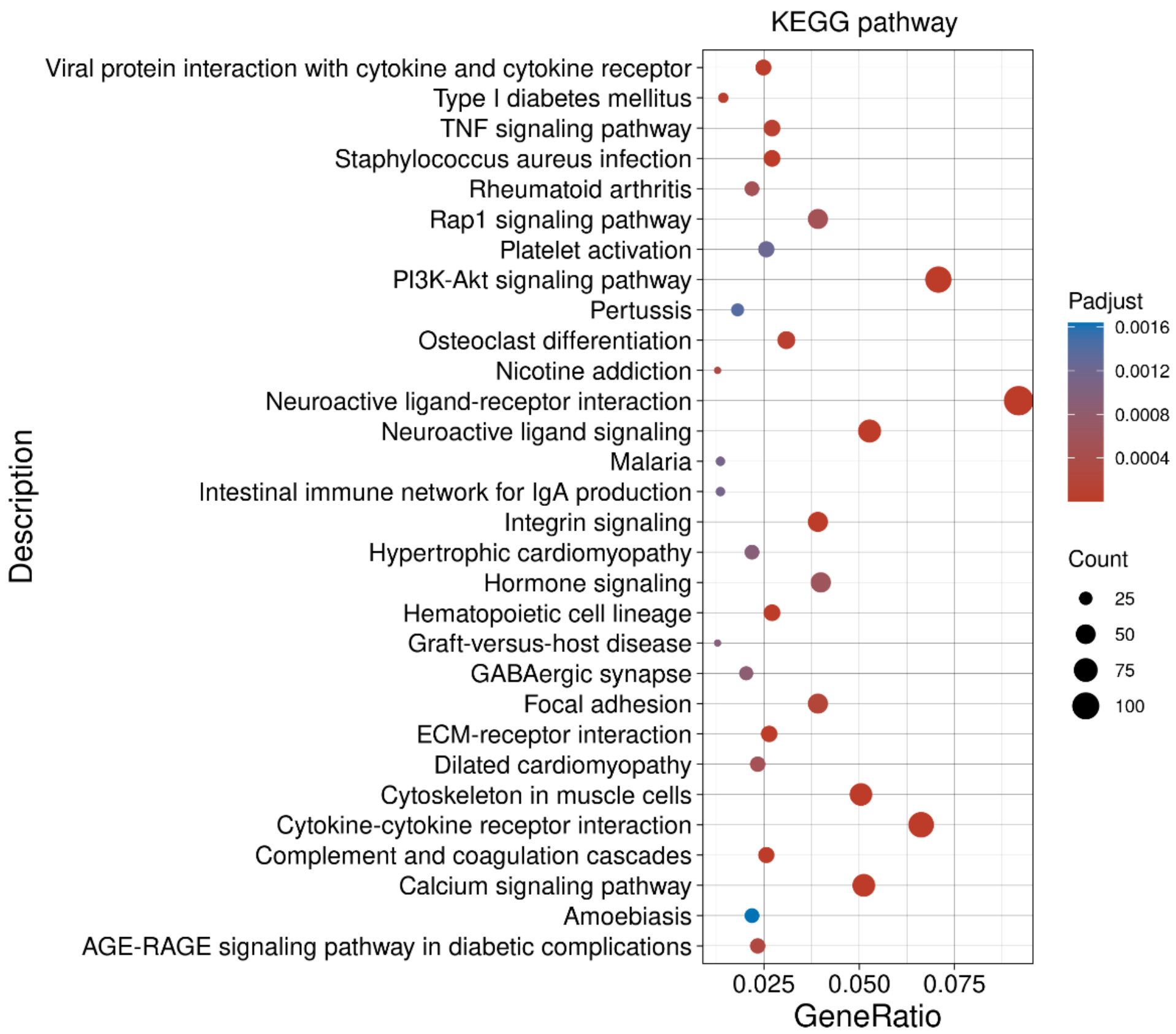

Supplementary Figure S2. KEGG pathway enrichment analysis of differentially expressed genes between TLE-HS and control hippocampal samples (GSE256068, 59 TLE-HS vs 11 controls). Over-representation analysis was performed using the enrichKEGG function in the clusterProfiler package in R. Differentially expressed genes were defined as those with adjusted $P < 0.05$ and $|\log_2 \text{fold change}| > 0.585$ (DESeq2). The complete enrichment results, including gene counts and statistical significance for each pathway, are provided in Supplementary Table S7. TLE-HS, temporal lobe epilepsy with hippocampal sclerosis.
